# Customizing delivery nano-vehicles for precise brain tumor therapy

**DOI:** 10.1186/s12951-023-01775-9

**Published:** 2023-01-28

**Authors:** Yang-Bao Miao, Wang Zhao, Gao Renchi, Ying Gong, Yi Shi

**Affiliations:** 1 Department of Haematology, Sichuan Academy of Medical Sciences & Sichuan Provincial People’s Hospital, School of Medicine of University of Electronic Science and Technology of China, No. 32, West Section 2, First Ring Road, Qingyang District, Chengdu, 610000 China; 2 Sichuan Provincial Key Laboratory for Human Disease Gene Study, Center for Medical Genetics, Sichuan Provincial People’s Hospital, University of Electronic Science and Technology of China, Chengdu, 610072 Sichuan China; 3 Natural Products Research Center, Institute of Chengdu Biology, Sichuan Translational Medicine Hospital, Chinese Academy of Sciences, Chengdu, 610072 Sichuan China; 4 Research Unit for Blindness Prevention of Chinese Academy of Medical Sciences (2019RU026), Sichuan Academy of Medical Sciences & Sichuan Provincial People’s Hospital, Chengdu, 610072 Sichuan China; 5 School of Life Science and Engineering, Southwest Jiaotong University, Chengdu, 610031 People’s Republic of China

**Keywords:** Nanoparticle, Drug delivery, Central nervous system, Tumor, Blood–brain barrier

## Abstract

Although some tumor has become a curable disease for many patients, involvement of the central nervous system (CNS) is still a major concern. The blood–brain barrier (BBB), a special structure in the CNS, protects the brain from bloodborne pathogens via its excellent barrier properties and hinders new drug development for brain tumor. Recent breakthroughs in nanotechnology have resulted in various nanovehicless (NPs) as drug carriers to cross the BBB by different strategys. Here, the complex compositions and special characteristics of causes of brain tumor formation and BBB are elucidated exhaustively. Additionally, versatile drug nanovehicles with their recent applications and their pathways on different drug delivery strategies to overcome the BBB obstacle for anti-brain tumor are briefly discussed. Customizing nanoparticles for brain tumor treatments is proposed to improve the efficacy of brain tumor treatments via drug delivery from the gut to the brain. This review provides a broad perspective on customizing delivery nano-vehicles characteristics facilitate drug distribution across the brain and pave the way for the creation of innovative nanotechnology-based nanomaterials for brain tumor treatments.

## Introduction

Tumor relapse caused by extramedullary infiltrate poses the biggest threat to patient survival [1]. The central nervous system (CNS) is one of the extramedullary places that cancer (acute leukemia and lymphoma) manifests itself in the most frequently [2]. It is estimated that between 2 and 10% of individuals with acute leukemia will experience an isolated CNS relapse despite the addition of systemic chemotherapy to the CNS-directed therapy and cranial irradiation in all patients [3]. Patients with central nervous system involvement have a prognosis similar to those who experience a recurrence in their bone marrow [4]. The mechanisms of CNS involvement are little known despite their therapeutic importance. An essential factor in this is the function of the blood–brain barrier (BBB), which serves to restrict the flow of substances between the bloodstream and the brain [5]. The central nervous system is so transformed into a safe haven for cancer cells and becomes a source of cells that seed extraneural locations. Clinical investigations have shown that the brain tumor recurrence rate is exceedingly high, despite the fact that current treatments for brain tumor typically include high-dose systemic chemotherapy, targeted medication therapy, and other strategies [6].

Several different strategys of therapeutic delivery have been developed in order to transport chemotherapeutic medicines across the BBB for the purpose of treating brain tumor [7]. These strategies were developed in order to improve treatment strategies that are used to control brain tumor while reducing the likelihood of backup adverse effects and neurocognitive sequelae [8]. Among them, the regulation of tight junctions in response to either chemical or physical stimuli as well as the alteration of drug molecules have revealed some promise [9]. The effectiveness of the drug transport mechanism may be enhanced by modulating tight junctions using a range of physical or chemical stimuli; however, exposure to high concentrations of these stimuli can have a deleterious effect on brain function [10]. Although modifying drug molecules through lipidation is an efficient method for crossing the BBB and allowing passive penetration of therapeutics, this tactic is only applicable to very small drug molecules (those with a molecular weight of less than 500 Da), which severely restricts its scope of application and availability [11]. Furthermore, because of the very selective character of BBB, the Trojan horse technique for carrying pharmaceuticals through BBB is extremely difficult to implement successfully [12]. Even if drug molecules are able to successfully pass through the BBB endothelium, there is still a chance that the drugs will be expelled directly into the bloodstream via the mechanism of the ATP-dependent efflux pump [13]. This is due to the presence of P-glycoprotein at the luminal cell surface, which is also known as multidrug resistance protein.

**Fig.1:**
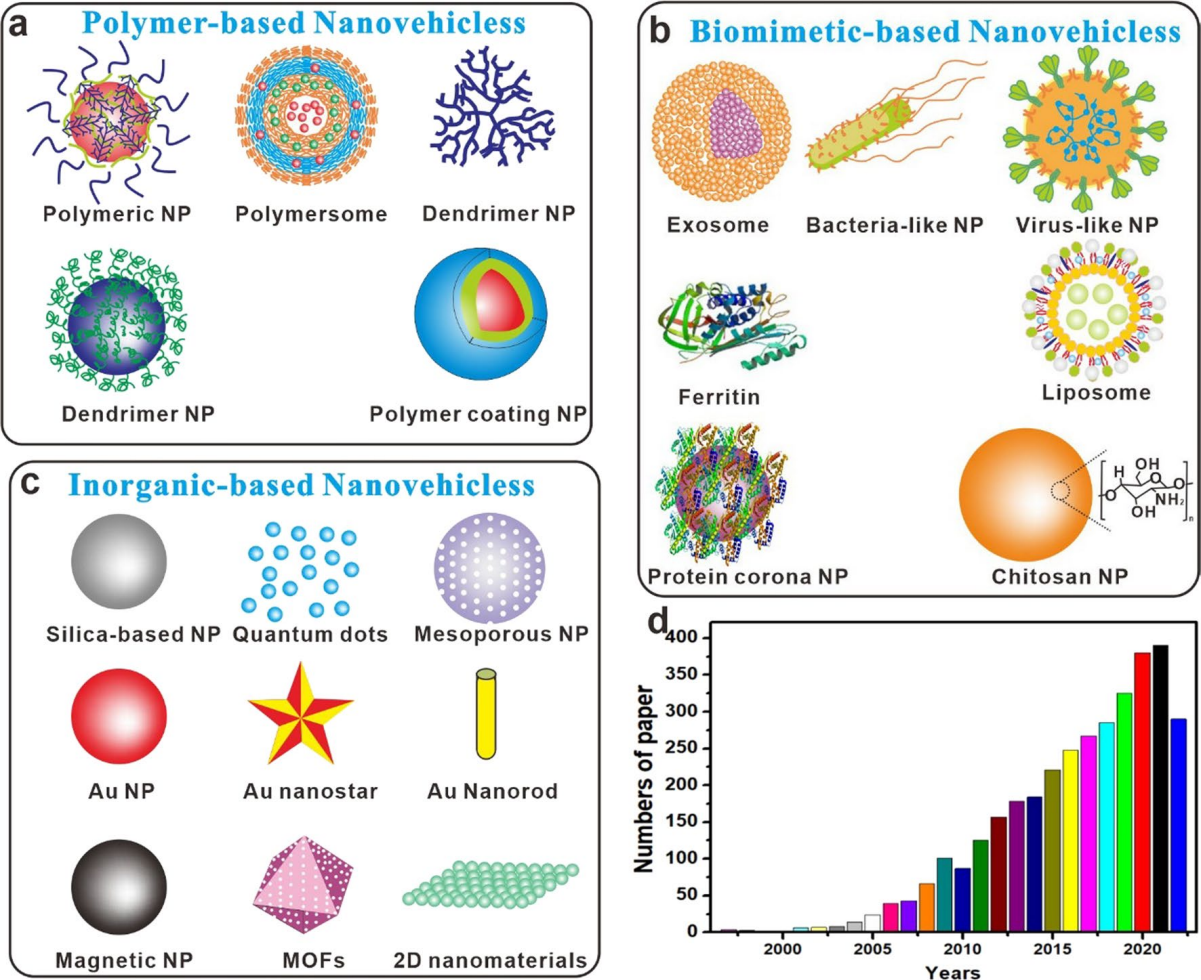
**a**, **b**, and **c** Schematic and structure of nanovehicless used to cross the BBB; **d** Annual frequency of scientific papers including the term inside the title

In recent years, one of the primaries focuses of research has been on the development of a method that does not involve any sort of intrusive procedure in order to transport medicines and macromolecules to the brain [14]. Since the introduction of nanotechnology, many different types of nanomaterials have been explored as prospective carriers due to their unique features for anti-brain tumor [15, 16]. These include their tiny size, high drug-loading capacity, ease of design, outstanding stability, biocompatibility, and biodegradability. Figure 1a, b, c shows some examples of these nanomaterials. For the delivery of drugs across the BBB without compromising its structure or functionality, nano-carrier-based transport approaches have emerged as a promising new dawn. In Fig. 1d, we see a breakdown of the number of publications published in each year on the topic of using nanovehicles (NV) to transport a drug, gene, or other therapeutic agent over the BBB. The ever-increasing number of research efforts in this field attests to the fact that NP-based drug carrier across BBB is not only a burgeoning academic area, but also has tremendous practical promise. This potential is indicated by the exponential growth of studies that have been conducted in this field.

**Fig. 2:**
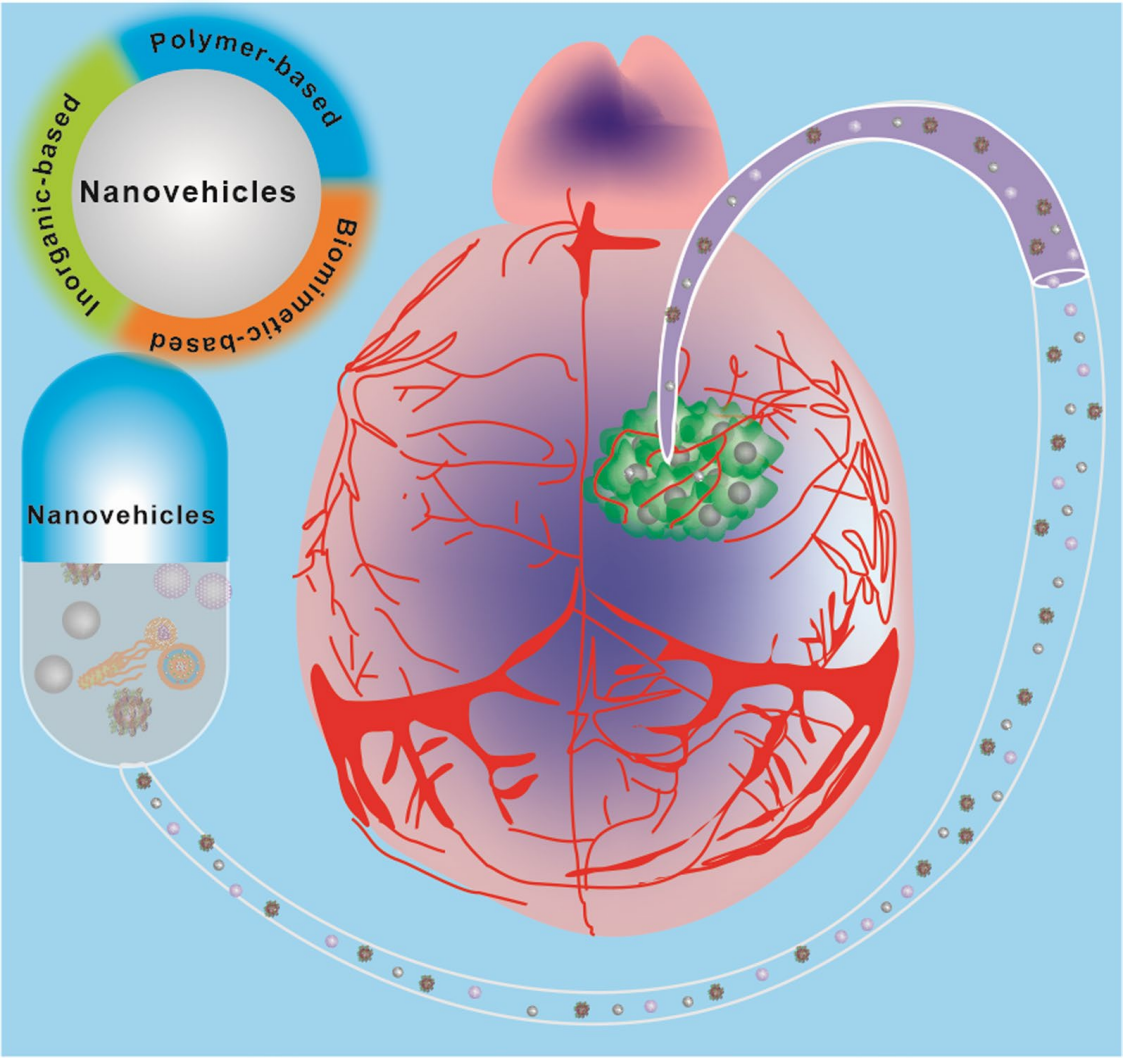
Customizing delivery nano-vehicles for precise brain tumor therapy

In this review, our primary focus is on providing a complete overview of the development and application of customizing delivery nanovehicles that are applicable across the BBB (Fig. 2). Though there have been numerous reviews on the topic of NP-mediated brain medicine delivery, the specific BBB characteristics, role of NPs, and their particular environment have only rarely been confirmed. As a result, we center our attention on the unique functions that NPs play in drug delivery across the BBB, the recent successes and accomplishments of nanovehicles-based drug delivery, and the potential for nanovehicles-based technologies to treat brain tumor in the future. In addition, a summary is provided of current developments in the understanding of the nature of the BBB, medications for brain illnesses, and various drug loading strategies.

## Pathogenesis of brain tumor

In the early 1970s, the issue of how tumor cells enter the central nervous system was brought up for the first time [17]. However, the examination of the ways by which tumor cells enter the CNS continues to be a challenging task because current model systems are unable to accurately portray the complexity of the central nervous system (CNS) [18]. In spite of this, new methods have emerged in recent years, painting a clearer picture of the anatomical pathways used by tumor cells to penetrate the central nervous system [19, 20].

**Fig. 3:**
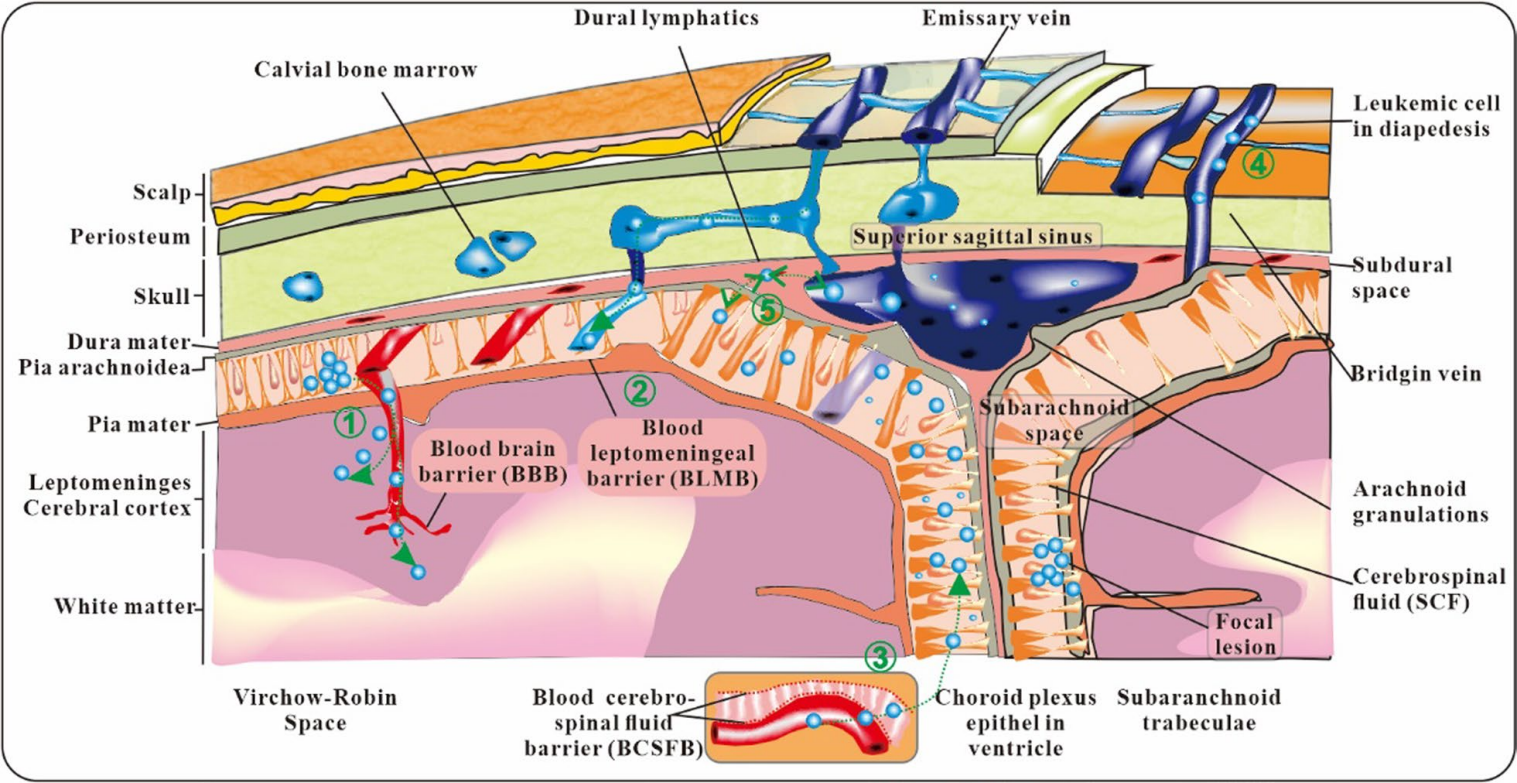
The route of tumor cells invades central nervous system

Anatomical structures can have a role to play in the tumor infiltration process [21]. Both the brain and the spinal cord are considered to be components of the central nervous system (Fig. 3). In physiological conditions, the interfaces between the structures of the central nervous system and the arteries form a complex barrier system [22]. This system is responsible for the selective and controlled entrance of substances and cells into the central nervous system [23]. In the context of tumor cell central nervous system (CNS) invasion, the blood-CSF-barrier (BCSFB), the blood leptomeningeal barrier (BLMB), and the endothelial blood–brain barrier (BBB) are thought to be the most significant barriers [24].

To form the BBB, the perivascular endothelial cells, astrocytes, and pericytes that surround the central nervous system parenchyma work together [25, 26]. The choroid plexus of the brain's ventricles is home to the BCSFB [27]. It is made up of epithelial cells inside the choroid plexus that communicate with one another through adherens junctions and vasculature in the meninges that are lined with fenestrated endothelium [28]. New studies have shown that the meninges contain a dural lymphatic system that drains macromolecules and cells from the central nervous system's deep substructure and works in tandem with the brain's vasculature [29, 30]. Therefore, the blood-dural lymphatics barrier (BDLB) may play a significant role in CNS invasion alongside the BCSFB, the BLMB, and the BBB.

Histopathological studies have shown that tumor cells can invade the central nervous system by growing along the Virchow-Robin spaces, which are tiny blood vessels that extend into the brain parenchyma, and eventually breaking through the pia-glial membrane and settling in the cortex [20, 31]. New findings detail the use of intravital microscopy to monitor the attachment and growth of a GFP-labeled Nalm-6 acute lymphoblastic leukemia (ALL) cell line [32]. The researchers determined that, similar to metastatic models of solid tumors, the injected cells become stalled in the microvasculature's branching structures soon after injection [33]. These results lend support to the idea that the parenchymal involvement of ALL, which occurs when tumor cells cross the BBB and enter the central nervous system, is probably of lesser. This suggests that the BLMB and BCSFB are the primary entry points for leukemia cells into the CNS.

Multiple in vivo studies employing xenograft models of ALL have successfully localized CNS invading cells in the subarachnoid region of the leptomeninges, which is near the ureteral venous sinuses [34]. This is in line with the results of the autopsy investigation conducted by Price and Johnson, who looked at 126 brain samples and found arachnoid involvement in 70 of them [35]. Initially, the leukemia cells only appeared in the superficial arachnoid and subarachnoid space [36]. Interestingly, in vivo studies have shown that Nalm-6 cells xenografted into NSG mice circulated through and briefly resided in the leptomeningeal vasculature, despite failing to cross the BLMB. Another finding that disproves the hypothesis that ALL enters the CNS via the BLMB and BCSFB was that tumor cells did not appear in the choroid plexus until late in the course of the disease. When investigating potential entry points other than the BLMB and the BCSFB, Yao et al. uncovered small cavities that spanned the bone marrow and the subarachnoid region [37]. They were thought to correspond to bridge veins since they co-stained for laminin and alpha-smooth muscle actin (SMA). Mice harboring the leukemia gene were found to have excessive numbers of tumor cells in these spaces. The highest laminin levels are seen on the abluminal (outside) surface of blood vessels. Yao and coworkers came to the conclusion that integrinlaminin-mediated activities were required for interaction with these arteries. This suggests that leukemia cells invade the subarachnoid region by a direct pathway along the membrane of bridging veins. Instead of taking the alternate route that involves the BSCFB, BLMB, and BBB. Additional studies are needed to verify if the CNS infiltration reported in individuals with ALL is consistent with the infiltration route observed in preclinical mouse models.

It was long thought that tumor cells traveled through the bloodstream to reach the brain and spinal cord [38]. While this is true, the recently found dural lymphatic system represents an alternative route of lymphocyte trafficking, and it appears that ALL cells may hijack the CNS lympha system to exit or enter the CNS [39]. This may be a promising new area of study with huge potential clinical applications. Due to the potential for tumor cells to enter and dominate the systemic circulation, this hypothesis takes on further weight in the setting of CNS relapse [40]. This could explain why patients with isolated CNS recurrence almost always present positive in the bone marrow and hence require systemic treatment. It is important to note that none of the research that have been described above demonstrate evidence for the uniqueness of any one entry. However, no matter how ALL cells penetrate the brain, nano-vehicles carrying drugs to the brain won’t restrict for brain tumor therapy [41].

## Pathways for transporting substances over the BBB

**Fig. 4:**
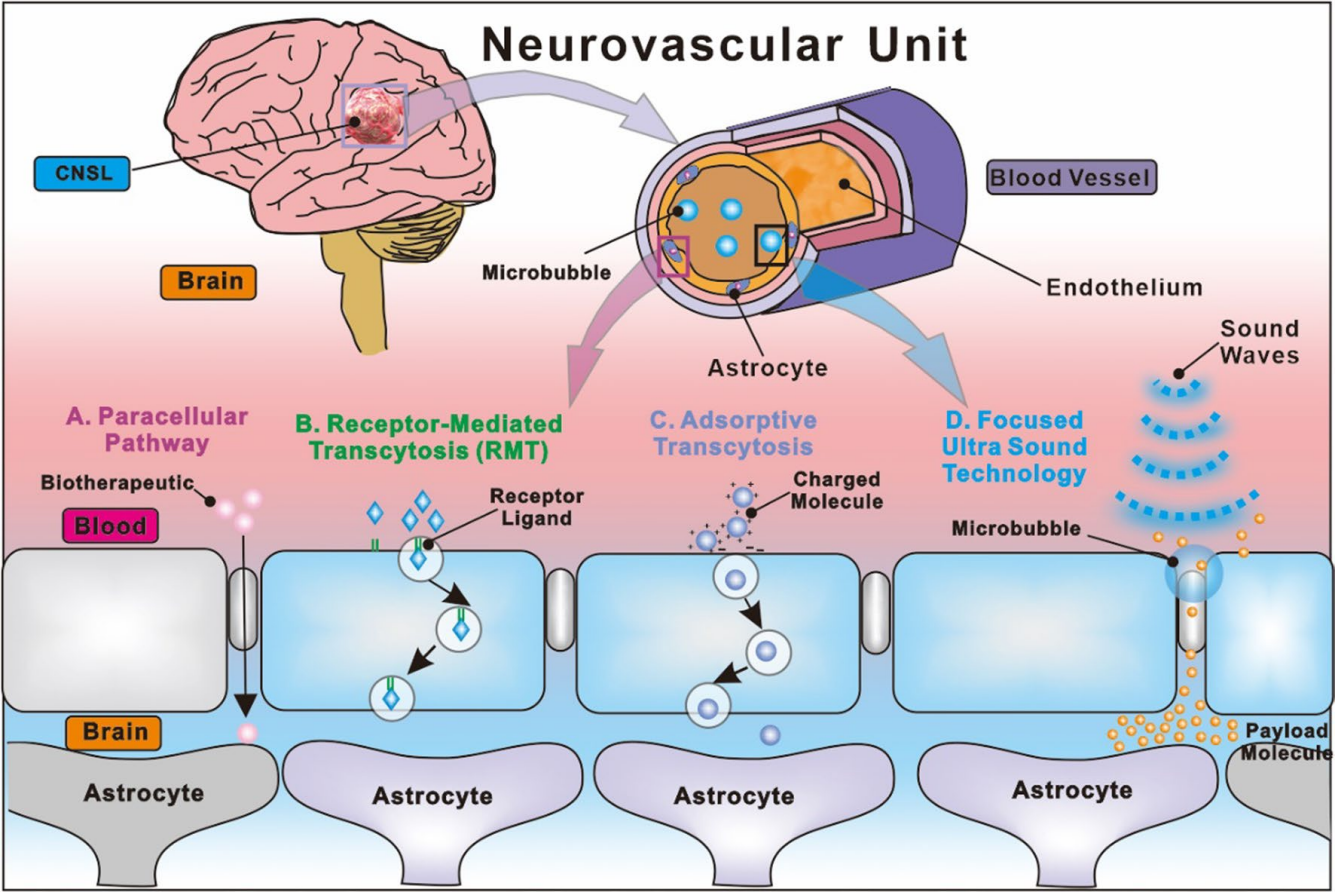
Nanovehicless transport pathways across the BBB

Multiple transport channels exist for the flow of proteins and peptides to maintain brain homeostasis, despite the BBB's role in preventing molecules from crossing from the brain parenchyma into the bloodstream [12, 42]. Diffusion-controlled transport processes including transcellular and paracellular transcytosis, receptor-mediated transcytosis, transporter protein-mediated transcytosis, cell-mediated transcytosis, and adsorbing mediated transcytosis (Fig. 4) [43, 44].

The process of transporting solute molecules over an intercellular space separating two endothelial cells that are adjacent is referred to as paracellular diffusion [45]. The positive concentration gradient that exists from the blood to the brain is what acts as the driving force behind this non-specific paracellular pathway. The paracellular space is only accessible to very small molecules that are water-soluble and have a molecular weight of less than 500 Da [46]. Researchers have discovered that modulations of tight junctions can lead to increased paracellular diffusion. On the other hand, the modification of the tight junctions may potentially enhance the permeability of the BBB to other chemicals that are undesirable [47]. “Transcellular diffusion” refers to the process by which solute molecules pass through an endothelial cell [48]. Through this pathway, the BBB can only be transported by a limited number of select small-sized chemicals that have the non-ionized materials, high hydrophilicity, and necessary lipid solubility [49]. In the same way as paracellular diffusion is driven by the positive concentration gradient, transcellular diffusion is driven by the negative concentration gradient [50].

Despite this, the ability of solutes to dissolve in lipids and to be hydrophilic assists in their passage through endothelial cells [51]. Hormonal steroids and alcohol, for example, can cross the BBB via transcellular diffusion, in which they dissolve into the plasma membrane of target cells [52]. Transcellular diffusion is another form of non-specific diffusion that works in a manner similar to paracellular diffusion [45]. Through a process known as active transport, certain transporter proteins, including the large amino-acid transporter (LAT) and the glucose transporter isoform Glucose transporter type 1 (GLUT-1), are able to transport molecules from across BBB [46]. Firstly, glucose or amino acids connect with blood-side transporter proteins so they can cross the BBB [47]. Amino acids and glucose enter the brain side of the cell due to a subsequent change in the structure of transporter proteins. Changing the medications so that they meet the structural binding criteria of the transporter proteins is crucial, however antibody conjugation on the drug surfaces is not required for this procedure. In addition, transporter proteins like GLUT-1 can only transport glucose over the BBB, limiting the potential of this route to be exploited for the administration of drugs [48].

**Fig. 5:**
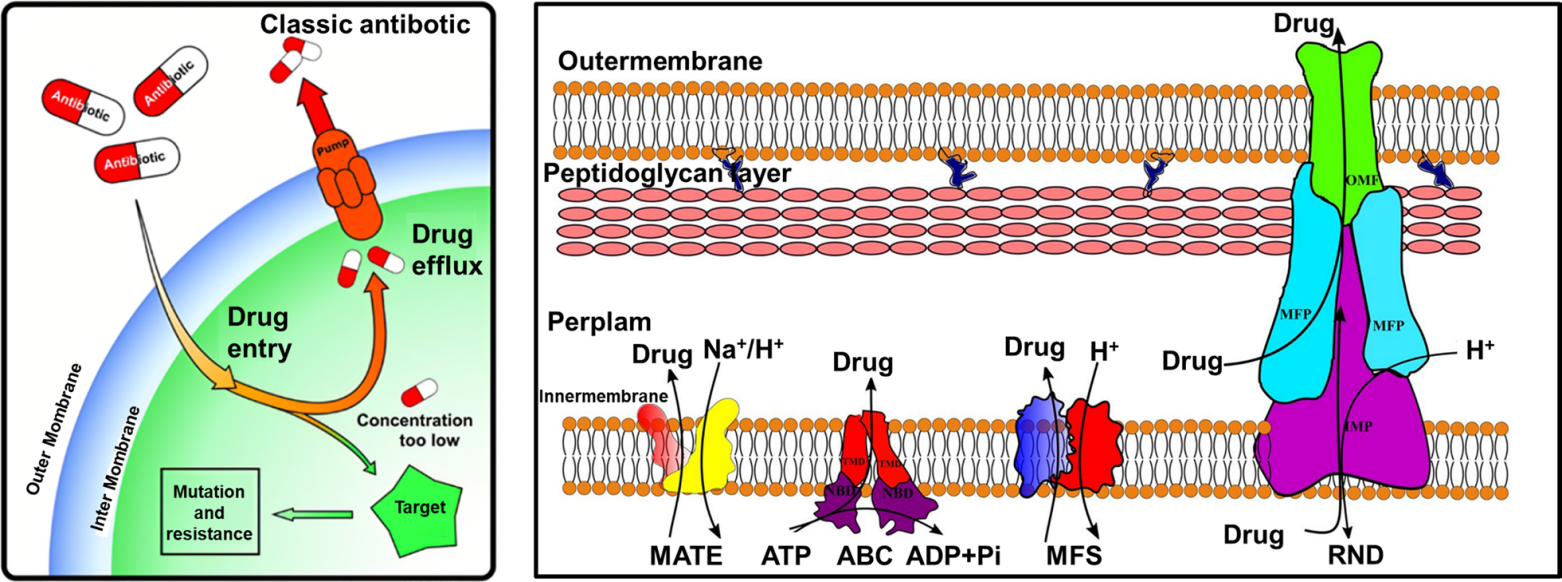
Mechanisms of resistance for multi-drug efflux pumps [53]

As was mentioned previously, the transport of medicines across the brain capillary endothelial cells is exceedingly challenging because of the strict properties of the BBB provided by the tight junction [49]. Figure 5 illustrates how the existence of efflux pumps further complicates the already challenging task of medication distribution [53]. Near the cell surface, or luminal side, of BBB endothelial cells. Efficient drug elimination is achieved by a family of proteins known as efflux pumps that includes pglycoprotein as well as the multidrug resistance proteins and breast cancer resistance proteins [51]. When working together, these proteins prevent the buildup of a wide variety of hydrophobic compounds and possibly harmful chemicals in the brain [52].

Additionally, the accumulation of medicines in the brain is stopped by these proteins in two distinct stages. In the first phase, they work together to stop endothelial cells from taking in drug molecules [54]. In the second phase, they actively remove anticancer drugs from the brain [55]. These drugs include doxorubicine, daunorubicine, and vinblastine, amongst others. It is generally assumed that ATP supplies the required power for the transport of medicines against a concentration difference that is negative [56]. These efflux pumps play both a negative and a positive role to make in BBB. For addition, they are accountable for lessening the neurotoxic and damaging impacts that medications have on the body. On the other hand, they place significant limitations on the dispersion of medicines in the CNS, which can be helpful in the treatment of CNS disease [57]. Therefore, altering efflux pumps at the BBB may be a useful strategy for increasing drug entry into the brain and providing new therapeutic options for a wide range of central nervous system (CNS) disorders.

## Nanovehicles for medication delivery across the BBB

There is an urgent requirement for the development of noninvasive drug delivery systems in order to address the increasing number of patients who are afflicted with CNS diseases. These strategies need to be able to mitigate the greater risk and cost factors that are associated with traditional surgical procedures, radiotherapy, and chemotherapy [58]. Transporting drugs or other molecules (including imaging agents, proteins, or nucleic acids) over the BBB in a way that doesn't disrupt normal brain function typically requires the use of a variety of nanocarriers delivery systems. In this review, we divide them into the three most frequent forms, which are nanovehicless based on inorganic materials, polymers, and biomimetic materials. Additionly, a selection of newly created nanoplatforms that are indicative of the field are emphasized in Table 1.

**Table 1:** Nanovehicles can cross the BBB, making them an ideal medication delivery system

Style	Nanovehicless	Drugs or agents	Drugs loading method	Size (nm)	Application	Methods of crossing BBB	Refs.
Inorganic materials	Gold NPs	–	–	3.5 ± 0.8	11-mercapto-1-undecanesulfate-coated	Protein corona NPs' evolution across the BBB	[59]
	Gold Nanostars	Ruthenium (II) complex	Electrostatic bonding	105	Pen peptide to enhance cellular internalization	preventing development of Ab fibrils	[60]
	CMC-coated Fe_3_O_4_ NPs	Dopamine hydrochloride	Covalent bond	14.05 ± 1.70	–	An agent for MRI and targeted drug delivery	[61]
	Ag NPs, TiO_2_ NPs, Ag^+^	–	–	Ag NPs 8 nm; TiO_2_ NPs 6 nm & 35 nm	Testing the permeability of the BBB in vitro	Ag NPs utilized ROS-induced cell death; exposure to Ag^+^ and TiO_2_ NPs damaged BBB via cytokine production. Testing BBB permeability in vitro	[62]
	Si NPs	Ruby dye	–	25, 50, 100	Evolution of Si NPs across the BBB	Graft from Lactoferrin	[63]
	MSNs	Resveratrol	Covalent bond	200		LDLR ligand peptide-functionalized CNS oxidative stress treatment	[64]
Polymers materials	Polymersome	Saporin Covalent bond	Saporin Covalent bond	76	Protein toxin delivery	Bonding ANG Protein toxin delivery	[65]
	Den-RGD	Cy5.5 Covalent bond	Cy5.5 Covalent bond	10	Photoacoustic shockwave therapy for the treatment of glioblastoma, in which CGS is used to stimulate the A2A adenosine receptor	Using CGS to activate the A_2_A adenosine receptor	[66]
	PLGA	3, 30—Diindolylmethane Encapsulation	3,30—Diindolylmethane Encapsulation	28–98	Creating the SSTR2 peptide Preventing glioma progression		[67]
	PEG–PLGA NPs	Paclitaxel Encapsulation	Paclitaxel Encapsulation	111.30 ± 15.64	Glioma therapy utilizing penetrating peptide (tLyp-1 peptide) as a coating	Decorating with penetrating peptide (tLyp-1 peptide)	[68]
	Liposomes	Doxorubicin Ammonium sulfate gradient loading method	Doxorubicin Ammonium sulfate gradient loading method	100 to 125	Chemotherapeutics for glioma therapy	Decorating with six peptides	[69]
	Amphiphilic Polymer-lipid NPs	Docetaxel Hydrophobic interaction	Docetaxel Hydrophobic interaction	100.1 ± 2.6	Treatment of brain metastasis of triple-negative breast cancer	Loading with PS 80	[70]
Biomimetic materials	VLPs	Ziconotide, an analgesic peptide that is produced by venomous marine snails, assembles itself	Venomous marine snail analgesic peptide ziconotide Self-assembly	54	Creating peptide therapies for use in the CNS	HIV-Tat peptide	[71]
	Lactoferrin NPs	HIV-Tat peptide Developing peptide therapeutics in the CNS	HIV-Tat peptide Developing peptide therapeutics in the CNS	70 ± 10	Treatment of cancerous Gliomas with the use of lactoferrin as a matrix	Using lactoferrin as a matrix	[72]
	Glucan NPs	Temozolomide or doxorubicin	Covalent bond	74.1 ± 3.8	gut-to-brain oral drug delivery for gliomas	Microphage-hitchhiked prodrug	[73]

### Polymer-based Nanovehicless

**Fig. 6:**
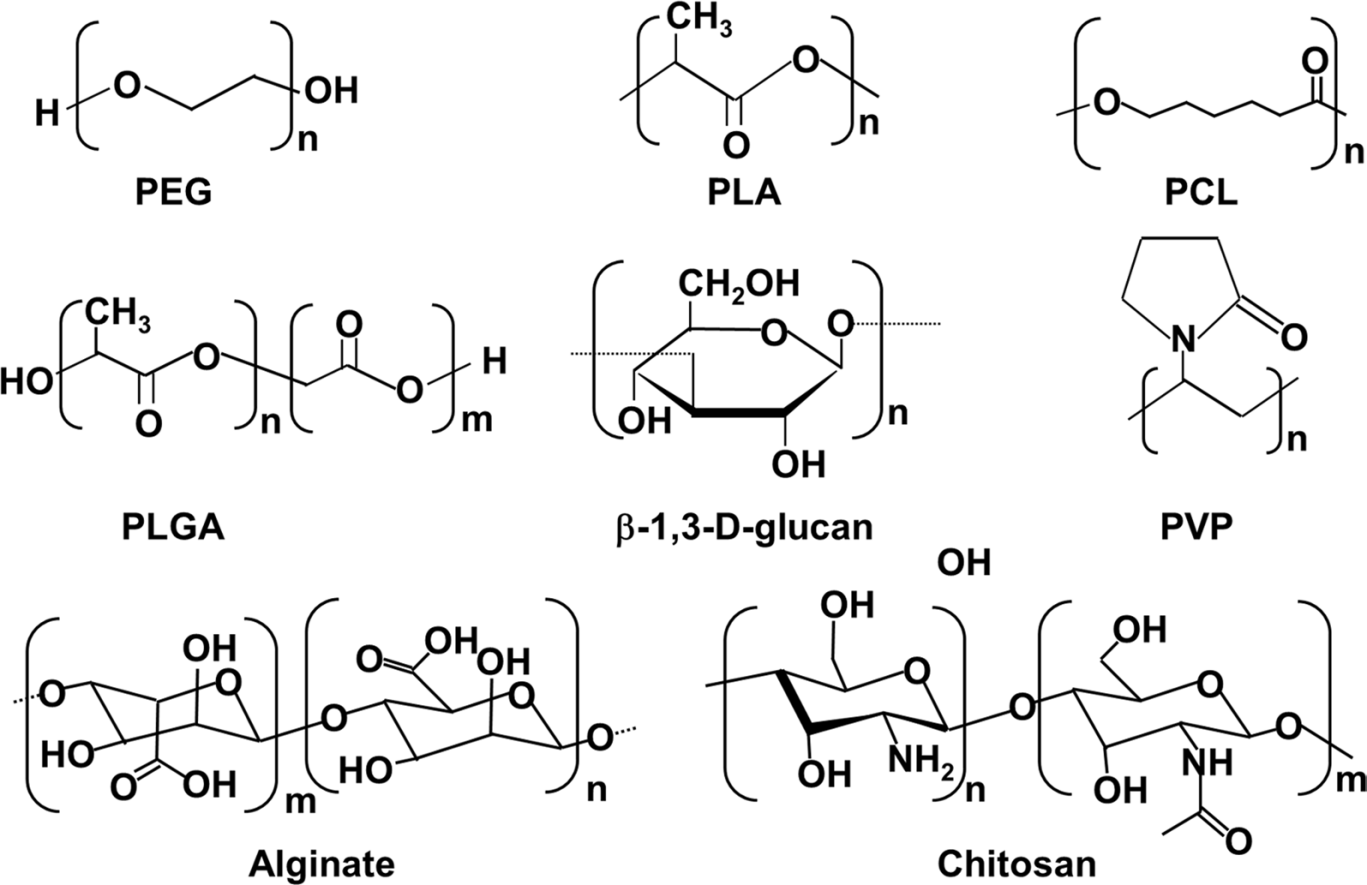
Chemical structures of different polymers

When it comes to transporting medications across the BBB, polymeric nanoparticles provide a number of benefits. For instance, they can increase the bioavailability of medications by lowering the rate of their breakdown by hydrolytic and enzymatic processes [74]. The utilization of drug-loaded polymeric nanocarriers makes it possible to achieve improved brain penetration as well as larger concentrations of medications within the tumor. There are four popular transport carriers that are based on polymers: polyethylene glycol (PEG), poly(lactic acid) (PLA) poly(lactide-coglycolic acid) (PLGA), β-1,3-D-glucan, PVP, alginate, and chitosan (Fig. 6). Among them, the PLGA nanoparticles have the advantages of lower toxicity, excellent biocompatibility, and carefully regulated drug release [75]. Using PLGA NPs as the delivery vehicle is one way to get over problems such as the insolubility of the drug and the absence of a passive transport alternative that can get the drug across the BBB. For the treatment of glioma, for example, Ghosh and colleagues have demonstrated the transport of PLGA nanoparticles through the blood–brain barrier (BBB) [76]. As part of their research, one-of-a-kind synthetic peptides that are targeted against somatostatin receptor 2 were grafted onto PLGA NPs. As a result, the NP transport capacity was significantly improved. In addition to the above, the findings demonstrated that this system was able to incorporate medicines within brain tumor and successfully trigger apoptosis. This form of drug carrier has the potential to reduce the cytotoxicity of the medications that it transports as a result of the superior biocompatibility of PLA and PEG NPs.

The traditional chemotherapy treatment for tumor does not provide clinical responses that are maintained over time. This disadvantage can be remedied by employing nanoparticles, which have the capacity to maintain the release of the medication that has been encapsulated or entrapped over a period of time, hence reducing the number of times that the drug needs to be administered. Among the polymers that are utilized for the manufacture of nanoparticles, poly (D,L-lactic-co-glycolic) acid, also known as PLGA, is the polymer that is most highly chosen due to the fact that it is biocompatible and biodegradable. Both the diffusion of the drug inside the polymer matrix and the breakdown of the polymer matrix contribute to the release of the medication at a pace that is maintained throughout time when it is encapsulated in PLGA. PLGA nanoparticles have been shown to deliver a prolonged release of all-trans retinoic acid, which has been shown to be useful in the treatment of tumor. The etoposide was loaded into the PLGA nanoparticles by a technique called solvent evaporation, which was followed by high pressure homogenization. Because these nanoparticles were able to keep releasing etoposide for as long as 72 h, it was hypothesized that they may be effective in the chemotherapeutic treatment of tumor. A modified version of the nanoprecipitation process was used to load the antimetabolite cytarabine into PLGA nanoparticles. Cytarabine is typically utilized for the treatment of tumor and brain tumor. The in vitro drug release from the pure medication was finished after two hours, whereas the release from the PLGA nanoparticles continued for up to twenty-four hours. It was hypothesized that decreasing the frequency of dosing with this continuous drug release of cytarabine would lead to a reduction in the adverse effects that are often associated with standard tumor treatment.

Additionly, a gatekeeping layer composed of biodegradable polyethylene glycol (PEG) and polylactic acid (PLA) was typically deposited on the surface of NPs in order to permit controlled medication release [77, 78]. For instance, Shen and colleagues used PLA as just a ROS-responsive linker to coating mesoporous silica NPs. This resulted in an improvement in the drug release even when subjected to high levels of oxidative stress. Because PEG has a reduced cell endosomal absorption, which can also slow the clearance of PEG-modified NPs, a thick PEG coating can help NPs spread passively in the brain [79]. This is due to PEG's decreased reticuloendothelial system accumulation. As a result, the PEGylation approach is used to change polymeric vectors in order to extend the amount of time that they spend circulating through the system, accomplish more effective penetration, and build up more in the brain. The researchers made use of these qualities of PEGs and covered the surface of the gold nanoparticles with PEG, as can be seen in Fig. 6a. Because of their inherent biocompatibility and biostability, they are able to traverse the BBB in both directions under normal conditions for an extended period of time. In addition to this, as a result of the acid-labile nature of cancer cells, they have the ability to rapidly dissolve within the cells of a brain tumor and to concentrate medications within the cancerous zone. Polymeric nanoparticles (NPs) have emerged as significant players in this industry; nonetheless, certain obstacles continue to impede their further expansion, prompting us to look for alternate solutions. Traditional linear polymers have a problem in that they have limited interaction sites and drug-loading regions.

Currently, some elegantly constructed polymeric NPs with huge specific surface areas are being used for drug delivery over the BBB. Dendrimers, for example, are a specific type of stretched polymer with much more tightly controlled structures. Dendrimer NPs, as opposed to traditional linear polymers, allow for the attachment of a substantially higher number of “peripheral” functional groups that may be regulated [80]. G5 polyamidoamine dendrimers were linked together with cyclic[RGDyK] peptide, CGS, PEG, and Cy5.5. This confers biocompatibility, BBB piercing capabilities, signal responsiveness, and tumor targeting on these polymeric NPs [81]. CGS, for example, can activate the A2A adenosine transporter, momentarily increasing extracellular space across brain capillary endothelial cells [82]. As a result, more NPs are able to cross over the BBB and spread into the brain side of the body. In furthermore, research has shown that increasing the number of generations of dendrimers has the potential to extend the amount of time that blood is circulated and to increase accumulation in the injured brain. This is a potential benefit that could be realized by increasing the number of dendrimers.

Unfortunately, one possible problem of these nanovehicless is that the majority of polymeric NPs are unable to track them in cells unless they are coupled to at least one fluorescent dye. This is a need that must be met in order for these carriers to be useful. This is a prerequisite for carrying out the action. Therefore, a complex manufacturing technique is necessary in order to attach fluorescent dye monitoring molecules to polymeric NPs. A brand-new kind of fluorescent polymeric NPs, based on poly [Triphenylamine-4-vinyl-(Pme thoxy-benzene)], has been developed by Lu and his colleagues (TEB) [83]. By doing so, we got rid of the time-consuming and difficult dye tracing method. Additionally, the functionalization of this nanoparticle with a variety of ligands, including lipoprotein, lactoferrin, and transferrin, resulted in an improvement in the transcytosis that takes place across the BBB. Furthermore, Lu and his coworkers have developed a completely original mathematical model in order to estimate the effectiveness with which TEB NPs are carried via BBB.

### Biomimetic-based nanovehicless

The immune system is able to quickly recognize exogenous NPs employed for drug administration, and the liver and kidneys are able to eliminate them from the body [84]. Since of this, the creation of biomimetic nanovehicless is gaining interest because these NPs can immediately detect and target ligand, continue to be circulating in the blood for an extended length of time, and prevent being killed by the immune system. Chitosan (CS), which is generated from chitin through the process of partial deacetylation, is considered to be a common biomimetic drug carrier due to its biodegradability, minimal immunogenicity, biocompatibility, and capability to access cellular tight junctions [85]. Chitosan (CS) is derived from chitin, and the process generates it [86]. In addition, several naturally occurring vesicles that are created with membranes, such as liposomes, exosomes, red cell membranes, or “Leukolike” coated nanoparticles, have been the subject of extensive research and study in the field of brain medication delivery as key biomimetic NPs. These vesicles include liposomes, exosomes, red cell membranes, and “Leukolike” coated nanoparticles. The fact that the phospholipid bilayer is the element that leads to its excellent biocompatibility is not something that should come as much of a surprise.

**Fig. 7:**
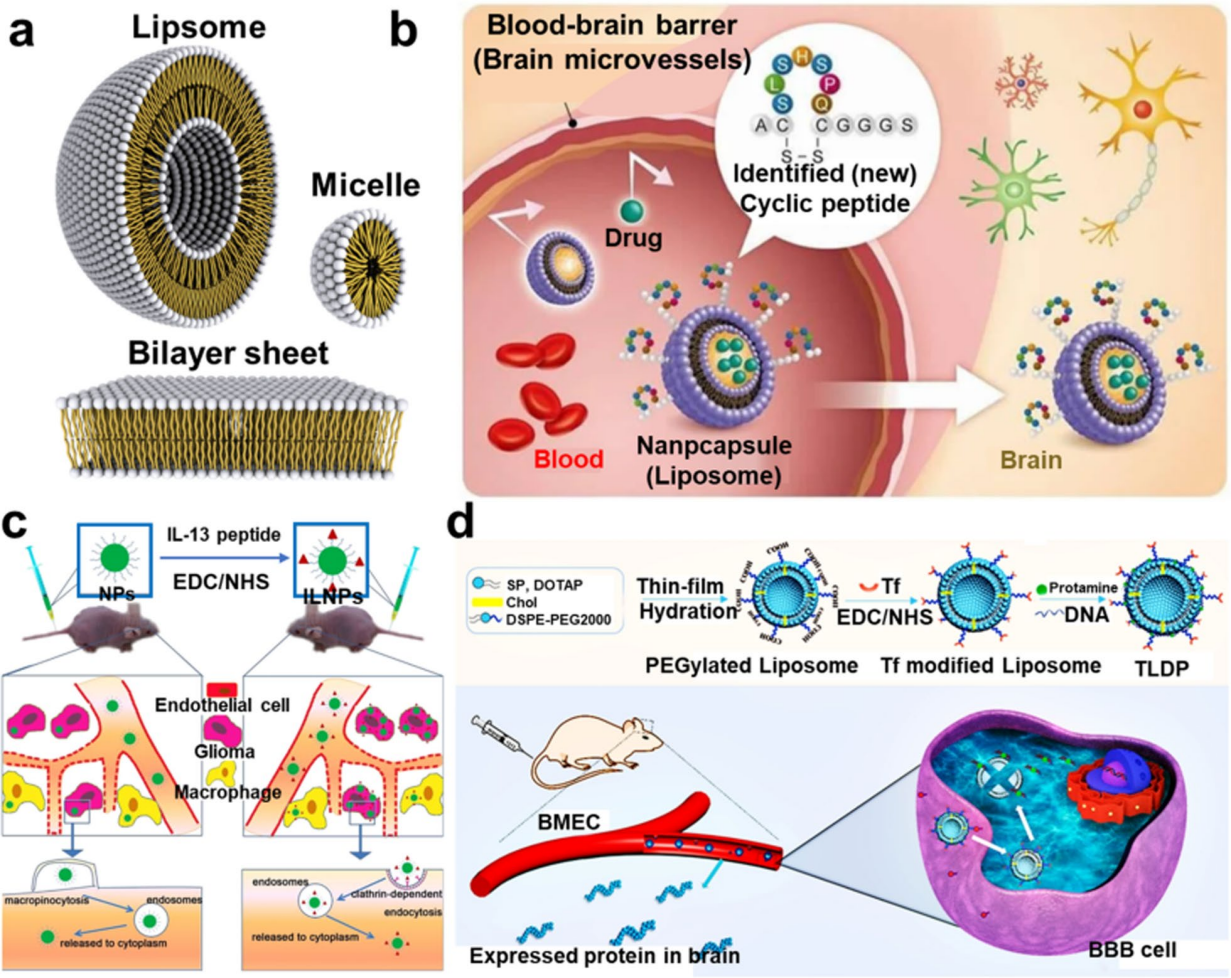
**a** The various morphologies of both a liposome-based drug delivery platorm [88]; **b** Using the recently discovered cyclic peptide, it is possible to transport pharmaceuticals by nanovehicles over the blood–brain barrier [91]. **c** Elucidation of brain cancer targeting methods of ligand-modified NPs [92]; **d** Mechanism of the gene delivery platform targeted to the brain [93]

The different morphologies of both a liposome-based drug delivery platform is depicted in Fig. 7a. [87] In this study, liposome nanoparticles were coupled with six different peptides in order to break through the BBB and deliver chemotherapeutics to treat brain tumor. According to the findings of the IVIS spectrum (Fig. 7b), peptide-modified liposomes may be able to pass through the BBB and increase the rate at which liposomes are internalized at the tumor site [88]. In addition, multifunctional proteins or proteins that self-assemble, such as the ferritin that is so often used, can be used to construct biomimetic nanovesicles that can be used for the delivery of drugs. Protein-based nanovehicles have the potential to boost cellular absorption since colloidal systems are one of the types of systems they belong to. Furthermore, these nanoparticles have many beneficial properties, such as the fact that they are non-toxic, non-antigenic, biodegradable, and simple to change on the surface. In addition, they can be easily fabricated.

A number of different medication delivery methods based on liposomes are currently undergoing clinical testing. The medication 2B3-101 is a PEGylated liposomal doxorubicin formulation coupled with glutathione. This formulation enables increased drug-delivery to the brain by employing specific transporters that are located on the BBB [89]. In 2011, individuals suffering from glioma or brain metastases as a result of breast cancer participated in a phase I/II clinical trial with the drug 2B3-101 (ClinicalTrials.gov identifier: NCT01386580). As a monotherapy and in combination with trastuzumab, a monoclonal antibody that interferes with the human epidermal growth factor receptor, the goal of the trial was to investigate the safety, tolerability, and pharmacokinetics of 2B3-101. Another delivery technology that is currently being investigated in clinical trials is a liposomal encapsulation of the camptothecin derivative and topoisomerase I inhibitor CPT-11 (ClinicalTrials.gov Identifier: NCT00734682) [90]. Patients with recurrent high-grade gliomas who are either wild type or heterozygous for the UGT1A1*28 gene are being recruited for the study as part of a phase I trial to investigate the efficacy, pharmacokinetics, and maximum dose that can be safely administered to these patients.

Protein-based nanovehicles have the potential to transport drugs that, after being given intravenously, are unable to cross the BBB. This is because of the properties described above (BBB). Figure 7c illustrates the study that the researchers undertook to investigate the stability of protein corona Au NPs as the particles were moving across the BBB [91]. In addition to candidates for vaccines and therapeutic treatments manufactured from protein-based nanomaterials, virus-like NPs (VLPs), which are a kind of noninfectious capsule protein-based NPs, have also been taken into account. VLPs are produced by a number of distinct types of viruses that assemble themselves. If you employ the protein that is found in the shell, you can use a technique that is often known as the Trojan horse to deliver encapsulated drugs or other agents. Exciting new study on engineered VLPs (as a nanocarrier) that can traverse the blood–brain barrier was presented by Anand et al. (Fig. 7d) [92]. They used the protein shell of the Salmonella typhimurium bacteriophage P22 as their starting material and used an endocytosis method to effectively deliver the analgesic marine snail amino acid ziconotide to an in vitro model of the BBB.

**Fig. 8:**
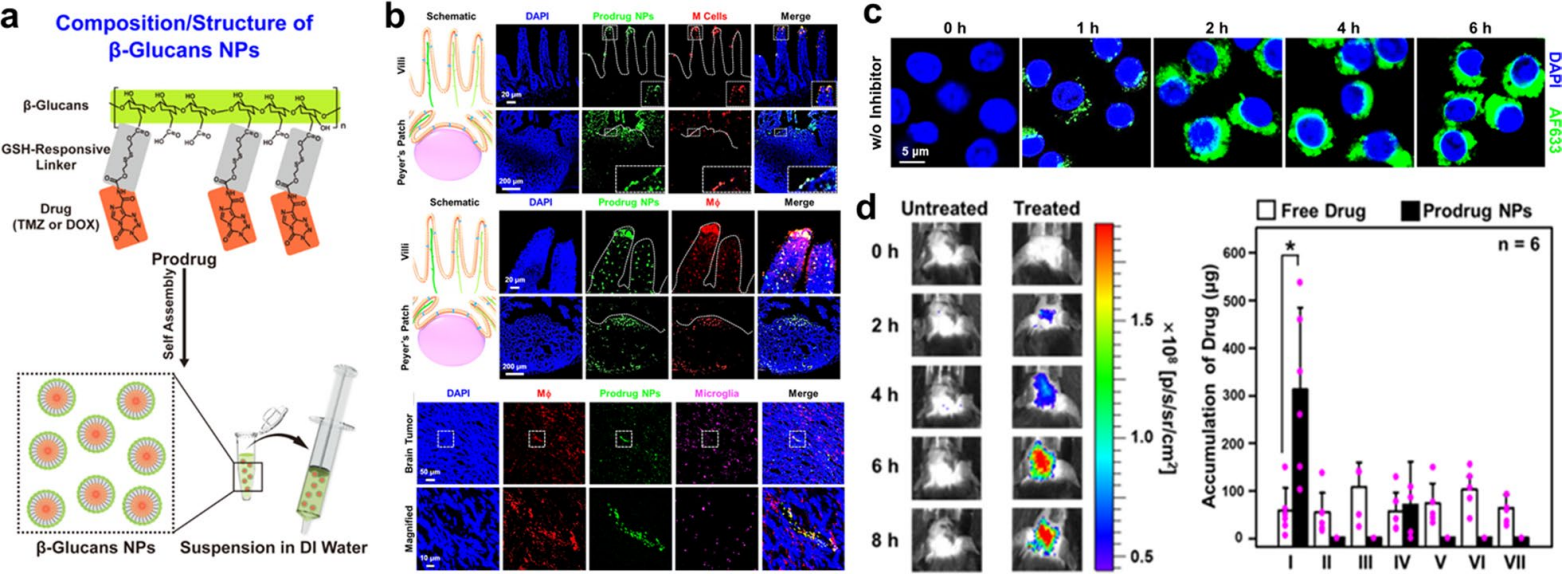
**a** β-glucans, a kind of prodrug, have a specific chemical makeup and structural arrangement [73]. **b** In ex vivo images, fluorescence-labeled prodrug nanoparticles were shown to be colocalized with M cells, macrophages, and the brain [73]. **c** Fluorescence-labeled prodrug nanoparticles being taken up by RAW264.7 macrophages in vitro (using the dectin-1 receptor) [73]; **d** Real-time IVIS pictures demonstrating accumulation of f-prodrug nanoparticles in brain tumor (ALTS1C1) collected at designated times following oral treatment to mice [73]. And quantities of the drug (TMZ) discovered in organs 6 h after therapy. I, the brain; II, the heart; III, the lungs; IV, the liver; V, the spleen; VI, the pancreas; and VII, the kidneys

Recently, an exciting technique for creating a gut-to-brain delivery platform without an active-targeted ligand has been developed. The strategy is described as follows: The yeast S. cerevisiae can create a wide variety of glucose polymers, one of which is called β-glucan [73]. Figure 8a demonstrates that the covalently prodrug is capable of self-assembling into NPs (β-glucans NPs) when it is placed in water [73]. A substance is referred to as a prodrug if, following its consumption, it passes through a process, either enzymatic or chemical, that results in the freeing of the pharmaceutically active drug molecule. When administered orally to a mouse model with an orthotopically produced glioma, β-glucans nanoparticles (NPs) selectively target M cells, pass through the barrier provided by the intestinal epithelium, and are endocytosed or hitchhiked by macrophages located in the periphery of the body (Fig. 8b) [73]. It is believed that β-glucans are microbe-associated chemical patterns that may be identified by immune-relevant cells (especially M cells and macrophages) whose cell membranes produce abundant amounts of the protein dectin-1 (Fig. 8c) [73]. β-glucans nanoparticles that have hitched a ride on macrophages are carried into systemic circulation via the ILS. Once there, they are able to break through the blood–brain barrier and ultimately end up in the brain tumor. In the end, the glutathione that is overproduced in the brain tumor cleaves the disulfide bonds in the β-glucans NPs, which frees the active drug and enables it to have its anticancer impact (Fig. 8d) [73]. Therefore, conjugating gut macrophage-targeting chemicals to oral delivery vehicles, such as glucans, may help in crossing the gut-to-brain barrier for the purpose of providing well-targeted treatment for brain tumors.

### Inorganic-based nanovehicless

**Fig. 9:**
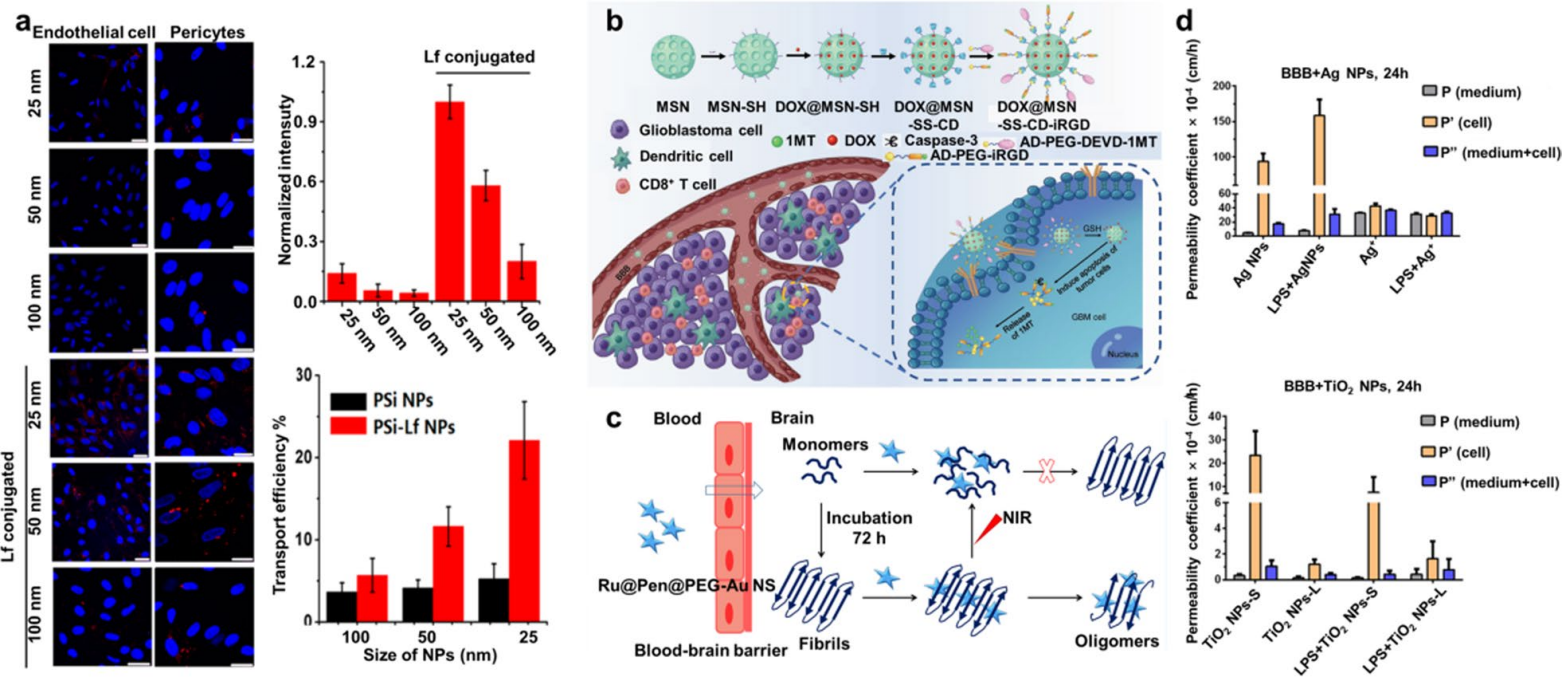
**a** Confocal images of an in vitro BBB model after incubation with represented for NPs size ranging from 25 to 100 nm [63]; **b** combined chemo-immunotherapeutic nanoparticles (DOX@MSN-SS-iRGD&1MT) through MSNs-based drug delivery system [96]; **c** Mechanism of tracking drug delivery (Ru@Pen@PEG-AuNS) for AD therapy [97]; **d** Permeability coefficient of AgNPs and TiO_2_ NPs are both used [98]

Inorganic nanoparticles have benefits over polymeric and biomimetic nanoparticles when it comes to medication delivery in the brain because of their size-dependent, different material, and great stability physicochemical features [94]. In the present day and time, diverse inorganic-based nanovehicless with a variety of architectures have been the subject of extensive research [13]. It is not difficult to modify inorganic-based nanovehicles by adding polymer or specific ligands in order to facilitate the dispersion of pharmaceuticals and macromolecules over the BBB. The Food and Medication Administration (FDA) of the United States has approved silica nanovehicles (also known as Si nanovehicles) for use in the food industry, making them one of the most promising options for drug delivery in the brain [95]. This is due to the fact that silica nanoparticles can be produced in a controlled manner, cost very little, and do not adversely affect living organisms. The Song group has created lactoferrin (Lf) modified Si-nanovehicles with the intention of exploring the size-dependent transport efficiency of Si-nanovehicless from across the BBB model (Fig. 9a) [63]. In order to lessen the amount of protein that binds to the surface of the Si Nanomaterials, polyethylene glycol was linked there. When compared to naked Si-nanovehicless, the Lf-attached Si-nanovehicless resulted in an increase in the transport efficiency across the BBB. In addition, Lf-modified Si-nanovehicless of varying sizes were investigated so that transport efficiency could be determined. The investigations showed that the best transfer efficiency was achieved with particles measuring 25 nm in size, which is approximately four times (21.3% higher) than that of bare Si-nanotechnology. We also compared the efficacy of Si-NP transport in monolayer (endothelial cell) and trilayer (fibroblast) BBB models (a coculture of astrocytes, pericytes, and endothelial cells). As another type of porous material based on Si, mesoporous silica nanoparticles, commonly known as MSNs, are becoming increasingly attractive for application in drug delivery systems. In addition to inheriting good biocompatibility, they possess a substantial specific surface area, which can be loaded with ligands or pharmaceuticals.

For the treatment of glioma, Kuang et al. investigated a drug delivery system based on traditional MSNs. The results of their study are shown in Fig. 9b. [96] Another type of inorganic material that possesses significant promise for use in medication delivery is gold nanomaterial. Under the conditions of near-infrared (NIR) laser irradiation, certain exceptional Au nanoparticles have the potential to convert photo energy into thermal energy, making them an excellent option for photothermal treatment (PTT). The fibrous Ab is a crucial part of Alzheimer's disease, and Yin et al. used Au-based nanovehicles, which are famous for their outstanding NIR absorption ability, to break it up. After being exposed to NIR irradiation, the fibrils system vanished in atomic force microscopy (AFM) and transmission electron microscope (TEM) pictures, demonstrating the vast surface area of Au-based nanovehicless for dissociating Ab fibrils (Fig. 9c) [97]. Additional compounds used to subvert the BBB include silver NPs and titanium dioxide NPs. An illustration of the passage of an Ag NPs, Ag ion, nand TiO_2_ NPs over an in vitro BBB model is provided in Fig. 9d. [98] Due to the magnetic characteristics of iron oxide nanoparticles, which reduce off-target effects when used as drug carriers, these nanoparticles are currently the subject of development and ongoing research. The research team led by Zhao produced a magnetic SiO_2_@-Fe_3_O_4_ nano-carrier, linked it to the cell-penetrating peptide Tat, and evaluated its fates in traversing the blood–brain barrier. Their experimental findings suggest that these particles, thanks to the cell-penetrating peptide Tat and the magnetic characteristics of Fe_3_O_4_, are able to successfully permeate the brain's endothelial cells. Inorganic nanoparticles (NPs) have been shown to have a number of potential adverse impacts on the structure and operation of BBB, despite the fact that they do offer a number of advantages. For instance, one research group investigated the potentially harmful effects of SiO_2_ nanoparticles (NPs) on the blood–brain barrier (BBB). They discovered that NPs could disrupt the structure of the BBB and cause inflammation in the BBB via ROS- and ROCK-mediated pathways. In conclusion, each NPs come with their own individual sets of benefits and drawbacks. For instance, the manufacture of inorganic nanoparticles still requires the use of organic solvents or inorganic reagents, both of which are quite pricey. Inorganic nanoparticles continue to be a major source of worry due to their toxicity as well as their slow clearance rate in living organisms. However, targeted effectiveness, big NP size, poor and manufacture difficulties still restrict them future usage in the brain medicine administration, despite their biodegradability, surface modification, and outstanding biocompatibility.

Moosavi and his colleagues employed nitrogen-doped titanium dioxide nanoparticles (N-TiO_2_) in conjugation with visible light [99]. They were able to demonstrate that this innovative NP-based photodynamic treatment (PDT) system induces both reactive oxygen species (ROS) and autophagy. The author demonstrated that well-dispersed photo-activated N-TiO_2_ NPs have the potential to promote terminal megakaryocyte differentiation or cell death in K562 tumor cells, and that this ability is dependent on the concentration of the NPs. These biological consequences are mediated by autophagy and are dependent on the ROS levels that are present inside the cell. In this scenario, low dosages of photodynamic therapy (PDT; 10 g/ml N-TiO_2_; 12 J/cm2) led to an increase in the levels of reactive oxygen species (ROS) and autophagy in PBLs, but it did not result in any growth-inhibiting or cytotoxic effects in the human normal-cell model. The author's combined N-TiO_2_ NPs and PDT technique allows preferential targeting and regulated photo-activated production of ROS and autophagy activation in tumor cells. This may offer a potential treatment approach for a broad spectrum of various cancer types.

Meanwhile, several different nanoparticle constructs that contain magnetic elements like iron, gadolinium, and manganese are either in the process of being developed or have already made their way into a clinical setting for the purpose of employing them as MRI contrast agents in the imaging of brain tumors. It has been demonstrated that exposure to these nanoparticles leads to an increase in signal enhancement over an extended length of time and improves one's ability to visualize the tumor border. As potential contrast agents for T2/T2* imaging of brain tumors, iron oxide nanoparticles have been the subject of substantial research [100]. In phase I clinical trials, patients with recurrent high-grade glioma who were receiving chemotherapy were given ferumoxytol, which is an ultra-small SPIO coated with polyglucose sorbitol carboxymethyl ether. Ferumoxytol was used as the MRI contrast agent along with a standard gadolinium chelate for these patients (ClinicalTrials.gov identifier: NCT00769093) [101]. In this dual agent MRI study using gadolinium and ferumoxytol, quantitative imaging changes of brain tumor vascularity after anti-angiogenic therapy with bevacizumab versus steroid therapy with dexamethasone are being evaluated. The steroid therapy with dexamethasone is being compared to the anti-angiogenic therapy with bevacizumab.

## Nanovehicle drug delivery parameter manipulation

**Fig. 10:**
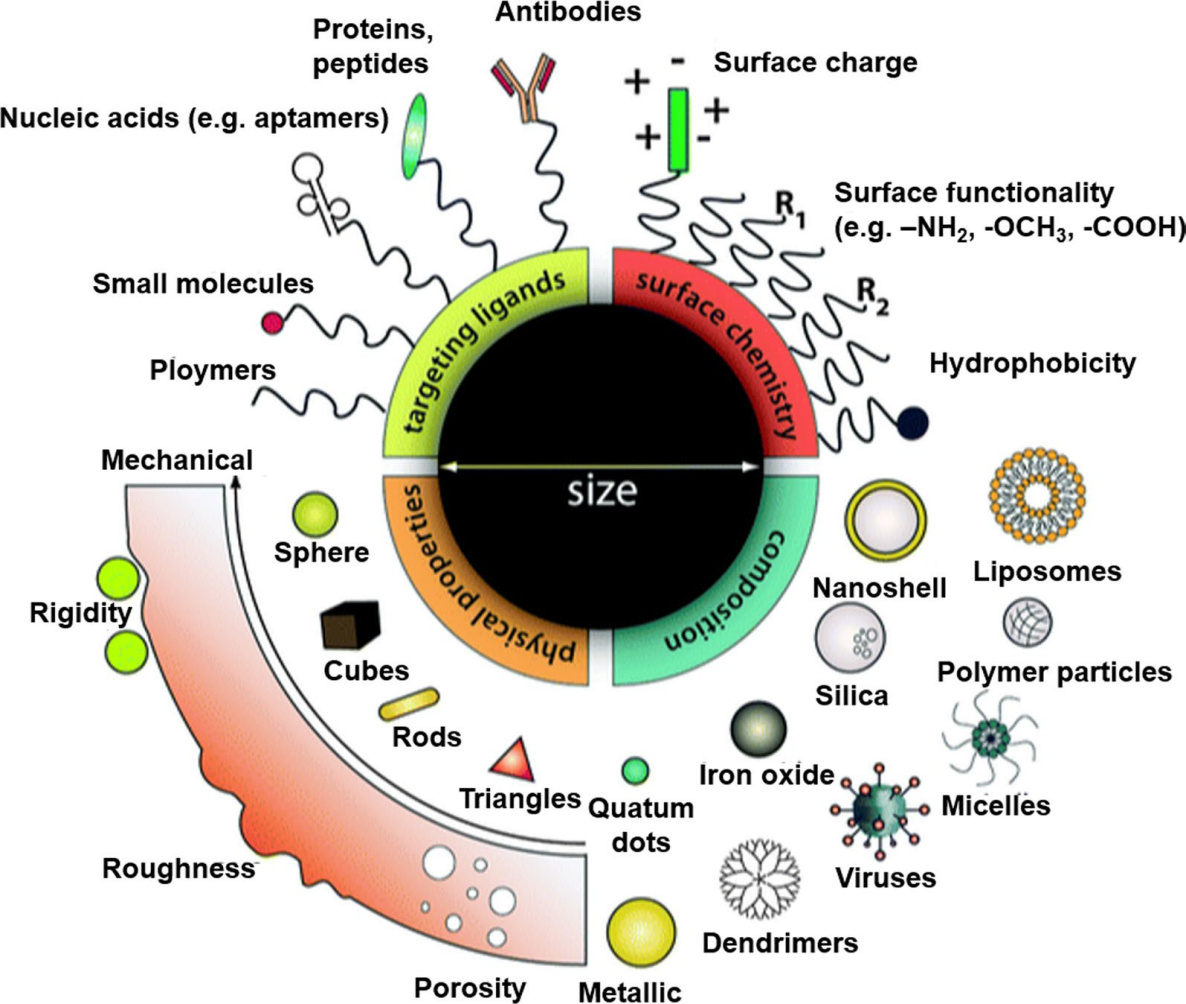
Strategies for the brain delivery of nanoparticles [99, 102]

Nanovehicle are currently attracting a lot of interest as a novel area of study in brain medicine delivery because of its various properties, such as mechanical attributes (lightweight, high flexibility), remarkable adaptability, and tunability to define the transport mode across the BBB. Nanoparticles' physicochemical properties are known to be strongly influenced by their surface chemistry aand shape. By adjusting nanovehicle ‘ physical characteristics (such as their surface charge, coating ligands and size, shape) (Fig. 10), it is possible to boost transport efficiency, enhance medication controllability, prevent RES, and increase therapeutic agent stability [102].

### Size

Medication efficacy upon brain delivery and nanovehicle transport through the BBB are often influenced by a variety of factors. One of the most important factors in nanovehicle intracellular localization and nanovehicle passage through the BBB is nanovehicle size [103]. Numerous studies, for instance, have suggested that receptor-mediated endocytosis makes it simpler for nanovehicle with a diameter of around 50 nm to be taken up by epithelial cells than uptake of other sizes of nanomaterials [104]. Another group investigated size-dependent changes in the permeability of silica nanovehicle using the BBB model (30, 100, and 400 nm, as well as the microparticles) [105]. The nanoparticles with a diameter of 30 nm were found to have the highest permeability coefficient of all the silica NPs, suggesting that the permeability of the BBB varies with particle size. Similarly, the photothrombotic ischemia (PTI) model showed that 30 nm biocompatible NIR NPs had a higher capacity for evaluating BBB damage than 10 nm and 60 nm nanovehicle. While smaller nanovehicle are capable of crossing the BBB, their rapid drug release and removal make them unsuitable for drug delivery. For the function of nanocarriers in the transport of medications to the brain, nanovehicle up to around 20 nm in size are often big enough to avoid renal excretion while yet being tiny enough to penetrate the BBB.

### Shape

The absorption of medications by cells can also be affected by the shape of the nanoparticles involved [106]. Over the course of the past few years, numerous nanovehicle configurations have been put through their paces in order to determine which one is most suited for treating brain diseases [107]. Shapes such as spherical, cubic, rodlike, and ellipsoidal nanovehicles are included in this category. Due to their convenience in preparation and surface modification, spherical nanoparticles can offer significant advantages over other nanoparticle shapes when applied to drug delivery. It has been demonstrated, on the other hand, that nanorods covered in particular antibodies have a greater capacity for adhesion than their spherical analogues do [108]. For instance, rod-shaped polystyrene nanovehicle coated with transferrin revealed a brain deposition that was seven times greater when compared to their spherical nanovehicle equivalents.

### Surface charge

Nowadays, the influence of surface charge on nanovehicles for medication transport across the blood–brain barrier has received an increasing amount of attention [109]. Because of the negatively charged structure of cellular membranes, zeta potential can have a direct impact on how much NPs are taken in by the cell. Therefore, the process of internalization of positively charged nanoparticles is considerably simpler than that of neutral or negatively charged nanoparticles. In addition to this, the surface charge of NPs is linked to a number of other important characteristics, including biodistribution of the particles and the half-life of their circulation in the blood. Alexis et al. discussed the elements that can determine the amount of time that NPs spend in the circulation as well as the organs in which they accumulate [110]. Negatively charged or neutral nanovehicle can reduce plasma protein adsorption and nonspecific cellular absorption, leading to a longer blood circulation half-life than positive charge NPs. Comparing the two NPs revealed this. Positively charged nanoparticles are poisonous, compromising the BBB. For brain medication delivery, negative zeta potential nanovehicles can avoid BBB disintegration. Zhang et al. linked peptide to lower NPs' zeta potential. This improved BBB transportation efficiency [111]. Poly(n-butyl cyanoacrylate) nanoparticles that were encapsulated with a negative charges (− 35.2 ± 1.1 mV) polysorbate 80 were found to have good stability and excellent transport through the BBB by a different set of researchers.

## Drug loading strategies

Given that this will affect the number of loaded medications as well as their binding strength, it is essential that the procedures for drug loading be both efficient and convenient [112]. Designing an ideal drug delivery system involves a number of steps. For this reason, it is absolutely necessary to have the very best interaction between the drugs and the nanoparticles that is possibly conceivable. It will be difficult to release the drugs if the interactions are either too strong or too weak; respectively, they will induce unneeded early leakage if they are too weak. If the interactions are too strong. A drug loading that is too low will have an effect on the therapy, while a medicine loading that is too high may cause certain adverse effects. Both of these factors might have an impact on the patient. Because of this, it is of the highest significance to determine whether or not the right binding between medications and nanocarriers has been achieved. Non-covalent adsorption, covalent bonding, and direct embedding are now the three techniques that are utilized the most frequently in the process of connecting various drugs used to treat CNS diseases with nanoparticles.

### Covalent bonding

The traditional method for connecting pharmaceuticals with nanoparticles is through the formation of covalent bonds. In the majority of instances, fast reversible condensation procedures consisting of ketals/acetal, boronate esters, and Schiff's base are utilized in order to carry out this method [113]. To give you an example, the process of dehydration condensation between NH_2_ and COOH allowed anticancer medications to be transformed on the surface of quantum dots [114]. Covalent bonding, on the other hand, is regarded a less versatile technique because there are only a limited number of reversible condensation reactions. In addition, the time it takes to attain the thermodynamic equilibrium is particularly long because strong covalent bonds produce slow binding and dissociation. As a result, it will take a very long time to complete.

Adsorption that is not covalent has lately emerged as one of the most frequent strategies for drug loading due to its simplicity of operation and the speed with which it may bind and transport pharmaceuticals [115]. Adsorption of medicines can be affected by a wide variety of non-covalent phenomena, including hydrogen bonding, halogen bonding, ion-ion electrostatic interactions, p-p stacking, van der Waals contacts, coordination bonding, hydrophilic, and hydrophobic properties. In the context of rational drug design, the halogen bond has been used as a strike to enhance drugtarget binding affinity in recent years. In other studies, scientists looked at the possibility of using several weakly-covalent contacts to firmly attach biomedicines to nanocarriers. It’s possible that this procedure will result in stronger bonds and more interaction sites than older approaches.

### Drug encapsulation

An alternate method for loading medicines is to place them in a vesicle that has been produced via a sealed lipid molecules membrane [116]. This method results in the pharmaceuticals being completely enclosed within the vesicle. In compared to both covalent and non-covalent techniques of drug immobilization, drug entrapment offers the ability to eliminate the risk of an unfavorable early drug-tissue interaction. In contrast to lipid nanovesicles, the approach of molecular design is used to directly entrap pharmaceuticals on the inside of the cavities of 3D nanomaterials. This has the potential to give customized molecularly controlled delivery systems. Tang et al. successfully used a molecularly imprinted polymer to entrap the drug aminoglutethimide and build a drug-delivery platform [117]. According to the findings of the experiments, this material managed to achieve both a high bioavailability and a speedy drug release rate.

### Ligands

Some laboratories are conjugating chemicals to polymeric NPs to boost the efficiency of brain drug delivery via the receptor-mediated route [118]. Polymeric NPs combined to targeted drugs improved the delivery of therapeutics to malignancies. Gint4.T is an aptamer that has been shown to target platelet-derived growth factor receptor b. Here, Lin and his team show that P NPs with the ligand Gint4.T attached may easily cross the blood–brain barrier (BBB) and accumulate in U87MG glioblastoma (GBM) cells [83]. Ligands for receptor-mediated transcytosis are commonly used to transport nanovehicle across the BBB. It has been shown that a variety of ligands, including as lactoferrin (Lf), transferrin (Tf), and low-density lipoprotein (LDL) receptors, may be used to selectively target receptors expressed on the BBB membrane and so promote receptor-mediated transcytosis. Conjugating ligands such as peptides, proteins, or antibodies to the surface of NPs is a common way to increase their targeting affinity with receptors. For BBB crossing via receptor-mediated transcytosis, several NP-based drug delivery methods rely on ligands. This article will discuss how various ligands may be categorized according to their capacity to facilitate BBB penetration.

### Utilization of ligands in the creation of protein corona

When nanovehicles are placed into a biological condition, the surface of the nanovehicles immediately begin to absorb proteins from the circulation. This process occurs almost instantaneously. A protein covering is produced as a consequence of this process and is called “protein corona” in this process [119]. More than seventy distinct serum proteins that are detectable in the bloodstream have been shown to be capable of adsorbing onto the surface of nanoparticles. In order to modify the Tween-80 on the surface of the NPs, Shubar et al. employed surfactant-assisted synthetic methods, and the findings Tween-80 modified NPs to efficiently pass BBB [120]. The Tween-80 treated nanocomposites showed significantly increased biocompatibility and absorption compared to uncoated NPs. Because these NPs are biocompatible, it is feasible to provide medication to the brain while experiencing significantly reduced cytotoxicity. This is made possible by the fact that these NPs are biodegradable. In addition to being biocompatible with the NPs, the protein corona has the potential to alter the surface chemistry of the NPs, provided that the proper designs are implemented. Because of this, there is a rise in surface avidity, which in turn leads to surface functionalization. As a direct result of this, there is also an increase in the efficiency with which drugs can be administered. Protein corona, on the other hand, has the potential to hasten the clearance of NPs from the circulation by means of the reticuloendothelial system (RES) [121]. This, in turn, reduces the quantity of NPs that are available for drug delivery to the brain and causes inflammation. Grafting nanoparticles with molecules of a surfactant can reduce the amount of surface fouling, which in turn lowers clearance and increases biocompatibility. For example, PEG treatment reduces NP opsonization and increases circulation time because of its antifouling properties, low surface charge, low ionic interactions, and hydrogen bonding. PEG modification is also very low in surface charge. Lipka and coworkers found that the half-life of NP was prolonged by a PEG chain of 10 kDa, which was previously unknown. The diameter of the PEG chain was used to calculate its length [122]. After twenty-four hours, they discovered that greater than 15% of the PEG-modified NPs had entered the bloodstreams of the mice participants. After keeping an eye on the mice for a full day, they came to this conclusion after noticing something interesting about their behavior. PEG grafting on NPs has the potential to effectively inhibit protein adsorption, which in turn slows down the clearance of NPs, which ultimately leads to a higher buildup of PEGylated NPs in the brain. As a result of this, PEG grafting on NPs has the potential to effectively inhibit protein adsorption.

### Utilization of ligands to target receptors on the BBB

Ligand-modified nanovehicles have a better capacity to react to receptors and to increase BBB permeability than nanovehicles that have not been changed in any way [123]. This is the case when comparing the two types of nanovehicles. Attaching transferrin peptide to nanoparticles, as demonstrated by research carried out by Ulbrich and his colleagues, makes it feasible to achieve well-surface dispersion despite the smaller particle size that is used. The tunable surface peptide has the ability to target the transferrin receptor on the endothelial cells that make up the BBB. This will cause the process of transcytosis to begin after it has been initiated. Only very recently have various other targeted ligands that are capable of attaching themselves to a wide array of receptors successfully been reported.

The monomers of amphipathic peptides play a crucial part in the process of enabling the uptake of NPs across the BBB, which in turn increases the efficiency of transport [124]. In general, amphiphilic peptide modified nanoparticles have a high affinity for the BBB and are stable. The energy penalty connected with peptide strands, especially increases undesirable interparticle electrostatic interactions, may be responsible for the increased stability. When it comes to transporting NPs via the BBB, the number of ligands present as well as the affinity of their receptors play a significant role (avidity). In mice carrying subcutaneous Neuro2A tumors, Choi and colleagues explored whether or not human transferrin (Tf) has an effect on the PEGylated gold nanoparticles (on tumor targeting) [125]. They discovered that a considerable proportion of the targeting ligands had an effect on the number of NPs that were found to be localised in cancer cells. The optimal ligand density for targeting brain microvascular endothelial cells and subsequent translocation across the BBB was identified by Moos et al. [126] When targeting the endothelial cells of cerebral blood vessels, the highest affinity is achieved at this optimum ligand density. Better dispersion and targeting of NPs for neurological disorders were also shown when they were modified with different ligands. Zhang et al. used a dual-targeting peptides ligand, TGN and QSH as ligands on PEG-PLA NPs, to treat neurological disorders [127]. TGN is a target ligand at the BBB membrane, whereas QSH has a high affinity for cells that are damaged by brain diseases. The NP with TGN and QSH was more effective in penetrating the hippocampus than the unmodified NP or the NP with only TGN added. Additionally, another team designed a Y-shaped liposome-based carrier that can traverse both the BBB and the BBB [128]. In vivo fluorescence imaging showed that liposomes coated with two ligands have better nanocarrier distribution in tumors than single-ligand-conjugated or unconjugated liposomes.

## Administration strategies for nanovehicles

If the administration strategies of these nanovehicles could (1) enable for specific distribution to and spread to the interior of the tumor and (2) minimize neurotoxicity and systemic toxicity, then the therapeutic potential of nanoparticles could be improved for clinical translation. This would be accomplished by improving the specific distribution of these particles [129]. In this article, we will go through the essential tactics for the administration of nanoparticles and evaluate the benefits and drawbacks of using them to treat brain cancer.

### Oral administration

Taking medications orally has many benefits, including reducing patient stress and improving their quality of life by removing the need for painful and potentially infectious injections [130]. As a result, the delivery of drugs from the gut to the brain is of utmost significance. The route of drug oral administration from the digestive tract to the brain, including the vagus nerve, the immune system, and blood circulation. However, the intestinal epithelial barrier (IEB) and the blood brain barrier (BBB) prevent the majority of orally delivered anticancer treatments from crossing into intracerebral diseased areas [73]. The BBB protects the brain from dangerous compounds in the bloodstream, while the IEB protects the digestive system from harmful viruses and toxins. Together, these two biological barriers significantly reduce the ability of orally delivered drugs to accumulate at the brain tumor site.

A prodrug with gut to brain drug delivery was created by Professor Sung [73]. The prodrug is conjugated onto the glucans using a linker that contains disulfide, which then results in the production of the prodrug. After oral treatment in mice that have glioma, the prodrug in its as-prepared form is able to selectively target intestine M cells, bypass the IEB, and be phagocytosed or hitchhiked by local macrophages (MΦ). The MΦ-hitchhiked prodrug is delivered into the circulatory system via the lymphatic system, allowing it to pass through the blood–brain barrier. Next, the glutathione that is overexpressed in the tumor cleaves the disulfide bond that is contained within the prodrug. This releases the active drug and increases the effectiveness of the treatment. According to these findings, the created prodrug has the potential to act as an oral drug delivery platform for the well-targeted therapy of gliomas, and it can do so by traveling from the gut to the brain.

### Nasal administration

The architecture, physiology and brain delivery pathway of the nasal cavity have been widely researched [131]. Basically, two regions of the nasal cavity, the respiratory region and the olfactory region, are important for medication absorption into brain or blood. Through the respiratory area mucosa some substances can enter the systemic circulatory system and subsequently cross the BBB to brain, while some can be immediately delivered to brain via the trigeminal nerve pathway or lamina propria adsorption from perivascular and lymphatic regions. By the olfactory mucosa chemicals can be transferred into the olfactory bulbs and then into cerebrospinal fluid through lamina propria absorption, olfactory neurons, lymphatic and perivascular spaces, and the trigeminal nerve pathway. Among these paths, the olfactory mucosa pathway is the most fast, and so it is the major conduit that facilitates drug delivery from the nasal cavity to the brain [131]. Nonetheless, the volume that can be intranasally delivered is relatively tiny (25–200 μL), which can limit the drug dose and the concentration of medication transferred into brain. The nasal cilial clearance further lowers the absorption period of medicine in the nasal cavity and drug metabolism and secretion can also limit the drug transfer into the brain.

Although there are already direct transport pathways to the brain in both the respiratory region and the olfactory region mucosa, the most important aspect of improving direct drug delivery to the central nervous system (CNS) via the nasal cavity is to increase the deposition and enrichment of drugs or their preparations on the olfactory mucosa, which will result in more direct diffusion of the drug from the olfactory mucosa to the brain [131]. This can be accomplished by increasing the concentration of drugs or as of right now, a variety of scientific methodologies have been created to improve the efficacy of drug transport from the nose to the central nervous system for the purpose of treating central nervous system illnesses. It has been proposed that novel approaches consist of combining a bioadhesive formulation with either an absorption enhancer or an active targeting mediated by an agglutinant or a brain-homing peptide derived from the screening of phage display libraries. This would be a novel way to attack the problem. In addition, iontophoresis, phonophoresis, electrotransport, and a number of other cutting-edge devices (Optinose ^™^, OptiNose UK Ltd., United Kingdom; DirectHaler TM, DirectHaler A/S Co., Denmark; and ViaNase ^™^, Kurve Technology, Inc., United States) have been used effectively.

### Intravenous administration

Systemic injection of nanoparticles is a particularly convenient method for delivery, as it enables repeated dosing to take place [132]. Even though there are many different nanoparticle systems now under research, the majority of them have the ability to target brain tumors via either passive or active targeting mechanisms. As was mentioned before, passive targeting takes place when nanoparticles are allowed to travel across a breached blood–brain barrier (BBB), which is referred to as the EPR phenomenon. In active targeting, the surfaces of nanoparticles are functionalized with targeting moieties that are unique to BBB and glioma cells.

Intravenous (IV) injections are an obvious choice for one of the delivery methods that can be used for nanoparticles [133]. The introduction of nanoparticles through intravenous means has been described in innumerable studies, including a significant number of the reports discussed above. In order to keep the tumor growth under control in the preclinical models, the nanoparticle-based therapies are frequently administered to the animals in various doses at an injection frequency ranging from once every three days to once every two weeks. The greatest dose that can be safely administered of a nanoparticle-based therapy is typically a sizeable multiple of that required for the free medication. In comparison to the free medications, the phase I beginning dose for these treatments is significantly lower when they are administered to patients every three to four weeks (ClinicalTrials.gov Identifier: NCT01386580) [134]. External factors such as a magnetic field or concentrated ultrasound can help capture systemically delivered nanoparticles at the site of a tumor. This is in addition to the tuning of nanoparticle size and surface features to effect intratumoral accumulation. The blood–brain barrier (BBB) can be locally disrupted using low-frequency focused ultrasound. Preclinical investigations have indicated that this method can safely boost the targeted delivery of therapeutic medicines into brain tumors. This breach of the BBB that was caused by ultrasound is only temporary and may be repaired; there is no permanent damage to the neurons or any other unfavorable long-term repercussions. Clinical trials have made use of magnetic targeting, another noninvasive method that aims to facilitate magnetic nanoparticle accumulation at a target region (ClinicalTrials.gov Identifier: NCT0005495, NCT00034333) [101]. The exposure of adults to magnetic field devices up to 8 Tesla and children to magnetic field devices up to 4 Tesla does not pose any safety issues, according to the guidelines provided by the FDA. Using mice afflicted with 9L-gliosarcoma, Chertok et al. revealed that it was possible to monitor the effects of intravenous administration of iron oxide particles using MRI. The authors observed that magnetic targeting led to a fivefold increase in the total exposure of glioma cells to the nanoparticles in comparison to non-targeted tumors, as well as a threefold improvement in the target selectivity for accumulation in the tumor as opposed to normal brain tissue. In spite of the fact that several methods have been established for the systemic administration of nanoparticles to circumvent the BBB, the overall percentage of systemically injected nanoparticles that are normally discovered in the brain is less than one percent. This non-specific accumulation of nanoparticles in normal tissues has the potential to induce significant deleterious consequences, as well as an increase in both mortality and morbidity among patients.

The most significant disadvantage of using systemic distribution is the possibility that nanoparticles will build up in organs that are not the intended targets, such as the liver, kidneys, spleen, and lungs. It is believed that nanoparticles such as iron oxide and gold nanoparticles are not hazardous to normal tissues; nevertheless, the long-term effects of nanoparticle deposition in the brain have not yet been fully investigated.

### Intracranial administration

One way to get around the BBB and avoid non-specific accumulation throughout the body is to administer nanoparticles locally, directly into a tumor location [135]. This is one of the available treatment options. Despite the fact that this mode of delivery, which can use either biodegradable or non-biodegradable polymers, has demonstrated some capacity to kill tumor cells, it is characterized by low drug penetration and has limits regarding dosing.

Convection enhanced delivery (CED), is another way for delivering nanoparticles to the intratumoral space, and it appears to circumvent these problems [101]. CED is a method that can be used to deliver therapeutic drugs directly to the location of the tumor, which has the added benefit of improving the distribution of molecules within tumor tissue. This technique makes use of pressure gradients to drive the bulk flow of nanoparticles, and agents are continually administered through the use of a catheter that is coupled to a syringe pump that can be inserted during surgery. In this scenario, it is possible to acquire larger drug concentrations and more broad distribution in a tumor in comparison to when the drug is administered systemically, all while experiencing minimal levels of systemic toxicity. CED of medicinal compounds has even made its way into clinical studies, and the same methodology can be applied to the administration of nanoparticles. In an intracranial U87 xenograft model, Noble et al. demonstrated that a single CED infusion of 1.6 mg nanoliposomal CPT-11 significantly prolonged median survival over 100 days [101]. This was in comparison to 28.5 days of survival when the free drug was administered or 19.5 days when the control liposomes were administered. In addition, the prolonged exposure to nanoliposomal CPT-11 did not exhibit any detectable toxicity to the central nervous system at any of the levels that were investigated. It has also been demonstrated that CED can deliver dendrimers and nanoparticles of iron oxide to brain tumors. Even while there is evidence that local administration is successful in treating brain tumors, there is still significant cause for concern due to the highly invasive aspect of this treatment approach.

## Future and outlook

The BBB is a significant barrier to the administration of medications used to treat brain tumors and other neurological disorders. This work provides a comprehensive analysis of the recent developments in nanovehicles-based drug carrier design for effective drug delivery strategies across the BBB. In our search for the most efficient means of drug delivery, we examined a wide range of delivery nanovehicles to learn more about the factors that affect penetration efficiency. However, it is important to note that several factors affect which nanovehicles are able to cross the BBB. Notable characteristics include size, shape, surface charge, ligand density, and drug loading method and delivery method (gut to brain delivery). Due to its distinct advantages, nano-vehicle based systems have been intensively investigated in an effort to create a synthetic platform for brain medicine delivery. However, there are still important questions that have not been well addressed. Moreover, several challenges must be surmounted before functional nanocarriers may be used effectively in medicine.

1. For usage in biomedical applications, the biodegradability and biocompatibility of nanovehicles are essential qualities; these characteristics can directly impact how far nanovehicles progress along the route to clinical translation. Although many studies have shown that biodegradable and biocompatible nanovehicles, such as inorganic nanovehicles, biomimetic nanovehicles, and polymer nanovehicles, can be transported across the BBB, the interactions between these nanovehicles and the immune system are complicated, and it is not known what effects, if any, they may have on human health. In comparison to other nanomaterials, certain polymeric nanovehicles exhibit higher levels of biodegradability and biocompatibility. This is an important point to keep in mind. More study is required to find a solution to the issue of the physiological stability of polymeric nanovehicles and to realize the objective of controlled medication administration across the BBB.
2. The surface charge of nanovehicles has a conflicting role to play in the process of bridging the BBB, and this role should be balanced. Cationic nanoparticles, which have a positive charge, are thought to have a greater chance of penetrating the blood–brain barrier (BBB) since endothelial cells have a complementary negative charge. However, the toxicity and half-life of anionic or neutral NPs are significantly lower than those of cationic nanovehicles. Furthermore, charge may lead to non-specific adsorption of protein or peptide in the circulatory system, which interferes with the normal operation of medicine administration. So far, the most successful method has been to encapsulate nanoparticles with polyethylene glycol (PEG) chains. This tactic leads to less nanovehicles endocytosis, lower macrophage uptake, and increased blood circulation.
3. There is a delicate balancing act to be performed when considering the competing roles played by the NPs' surface charge and the BBB-bridging process. It is difficult to find biocompatible nanocarriers that are also suited for controlled medication loading and release. In addition, only a limited number of medications was able to be administered to the brain tumor because of drug leakage that occurred during delivery.

Drug carriers based on nanoparticles should ideally have a high specific surface area and strong interactions with the drugs they are transporting. Drugs may be loaded into responsive porous materials to an extreme degree, and the materials can then release the drugs in a regulated fashion just where they’re needed most (in the sick tissue). Nanotechnology offers new possibilities for the creation of nano-carriers, which are essential for delivering drugs to their destinations. Nowadays, researchers are working on both multifunctional theranostic nanoplatforms and specialized nanocarriers that can traverse the blood–brain barrier. Computed tomography, photoacoustic imaging, the second NIR window optical imaging, and NP-based magnetic resonance imaging are all examples of such nanoplatforms. While still in the research and development phase, ligand conjugated nanoparticles have shown the most promise in transporting medications over the BBB.

Nano-vehicles have demonstrated a significant amount of potential and diversity in terms of their ability to encapsulate many chemicals simultaneously in controlled drug-delivery systems and direct them to the most inaccessible parts of the brain in order to inhibit tumor growth. Treatment options for other brain illnesses (e.g. stroke, AD and PD) are also restricted by BBB, just as they are for brain tumors. The development of nanoparticles can prove useful in the treatment of disorders of a similar nature. Furthermore, a growing number of studies are revealing that NPs have a healing effect on animal models of neurological diseases (e.g. stroke, AD and PD). It is necessary to conduct additional research in order to gain a better understanding of the differences in nano-vehicles transport between healthy animal models and diseased animal models. However, it is important to keep in mind the constraints of an experimental model, as such a model cannot perfectly simulate a particular human disease. Even though it is common knowledge that the BBB properties are significantly changed in in vivo models of Parkinson's disease, Alzheimer's disease, or stroke, no comprehensive research has been conducted to investigate how the physicochemical properties of nano-vehicles influence the transport and localization of NPs in the brain. To the best of our knowledge, there is not a single nano-vehicle formulation that is now being investigated in clinical trials for the treatment of stroke, Alzheimer’s disease, or Parkinson's disease. However, we can make the educated guess that it is only a matter of time before nano-vehicles generated in preclinical studies are examined in future clinical assays. Simultaneously, this review is also concerned about the gut to brain drug delivery system, which transports pharmaceuticals to the brain via the gut-brain axis. Thus, we anticipate that medication delivery via nanovehicles into the brain will have a bright future in the treatment of brain tumor illnesses.

## Data Availability

Not applicable.

## References

[1] Zhu D, Li Y, Zhang Z, Xue Z, Hua Z, Luo X, Liu Y (2021). Recent advances of nanotechnology-based tumor vessel-targeting strategies. J Nanobiotechnol.

[2] Dellacherie MO, Seo BR, Mooney DJ (2019). Macroscale biomaterials strategies for local immunomodulation. Nat Rev Mater.

[3] Zhang X, Chen J, Wang W, Li X, Tan Y, Zhang X, Qian W (2021). Treatment of central nervous system relapse in acute promyelocytic leukemia by venetoclax: a case report. Front Oncol.

[4] Cao HY, Tao T, Shen XD, Bai L, Wan CL, Wu DP, Xue SL (2022). Efficiency of anti-VEGF therapy in central nervous system AML relapse: a case report and literature review. Clin Case Rep.

[5] Huang CW, Chuang CP, Chen YJ, Wang HY, Lin JJ, Huang CY, Huang FT (2021). Integrin α2β1-targeting ferritin nanocarrier traverses the blood–brain barrier for effective glioma chemotherapy. J Nanobiotechnol.

[6] Ho JS, Zhang Y (2022). Wireless nanomedicine for brain tumors. Nat Nanotechnol.

[7] Vanner RJ, Dobson SM, Gan OI, McLeod J, Schoof EM, Grandal I, Dick JE (2022). Multiomic profiling of central nervous system leukemia identifies mRNA translation as a therapeutic targetblocking translation to target B-ALL CNS disease. Blood Cancer Discov..

[8] Zhang S, Zhang S, Luo S, Tang P, Wan M, Wu D, Gao W (2022). Ultrasound-assisted brain delivery of nanomedicines for brain tumor therapy: advance and prospect. J Nanobiotechnol.

[9] Zhang Y, Yang WX (2016). Tight junction between endothelial cells: the interaction between nanoparticles and blood vessels. Beilstein J Nanotechnol.

[10] Jia X, Yuan Z, Yang Y, Huang X, Han N, Liu X, Lei H (2022). Multi-functional self-assembly nanoparticles originating from small molecule natural product for oral insulin delivery through modulating tight junctions. J Nanobiotechnol.

[11] Liu B, Yan W, Luo L, Wu S, Wang Y, Zhong Y, Wang G (2021). Macrophage membrane camouflaged reactive oxygen species responsive nanomedicine for efficiently inhibiting the vascular intimal hyperplasia. J Nanobiotechnol.

[12] Mitusova K, Peltek OO, Karpov TE, Muslimov AR, Zyuzin MV, Timin AS (2022). Overcoming the blood–brain barrier for the therapy of malignant brain tumor: current status and prospects of drug delivery approaches. J Nanobiotechnol.

[13] Shilo M, Sharon A, Baranes K, Motiei M, Lellouche JPM, Popovtzer R (2015). The effect of nanoparticle size on the probability to cross the blood-brain barrier: an in-vitro endothelial cell model. J Nanobiotechnol.

[14] Wolak DJ, Thorne RG (2013). Diffusion of macromolecules in the brain: implications for drug delivery. Mol Pharm.

[15] Wang T, Zhang H, Qiu W, Han Y, Liu H, Li Z (2022). Biomimetic nanoparticles directly remodel immunosuppressive microenvironment for boosting glioblastoma immunotherapy. Bioactive materials.

[16] Yang M, Li J, Gu P, Fan X (2021). The application of nanoparticles in cancer immunotherapy: targeting tumor microenvironment. Bioactive materials.

[17] Aur RJ, Simone J, Hustu HO, Walters T, Borella L, Pratt C, Pinkel D (1971). Central nervous system therapy and combination chemotherapy of childhood lymphocytic leukemia. Blood.

[18] Zhang Y, Guo P, Ma Z, Lu P, Kebebe D, Liu Z (2021). Combination of cell-penetrating peptides with nanomaterials for the potential therapeutics of central nervous system disorders: a review. J Nanobiotechnol.

[19] Zhou F, Wen Y, Jin R, Chen H (2019). New attempts for central nervous infiltration of pediatric acute lymphoblastic leukemia. Cancer Metastasis Rev.

[20] Yao H, Price TT, Cantelli G, Ngo B, Warner MJ, Olivere L, Sipkins DA (2018). Leukaemia hijacks a neural mechanism to invade the central nervous system. Nature.

[21] Elbahlawan L, Galdo AM, Ribeiro RC (2021). Pulmonary manifestations of hematologic and oncologic diseases in children. Pediatr Clin.

[22] Kitchen P, Salman MM, Halsey AM, Clarke-Bland C, MacDonald JA, Ishida H, Bill RM (2020). Targeting aquaporin-4 subcellular localization to treat central nervous system edema. Cell.

[23] Wang J, Rong Y, Ji C, Lv C, Jiang D, Ge X, Fan J (2020). MicroRNA-421–3p-abundant small extracellular vesicles derived from M2 bone marrow-derived macrophages attenuate apoptosis and promote motor function recovery via inhibition of mTOR in spinal cord injury. J Nanobiotechnol.

[24] Stewart DJ, Keating MJ, McCredie KB, Smith TL, Youness E, Murphy SG, Freireich EJ (1981). Natural history of central nervous system acute leukemia in adults. Cancer.

[25] Tian X, Fan T, Zhao W, Abbas G, Han B, Zhang K, Xie Z (2021). Recent advances in the development of nanomedicines for the treatment of ischemic stroke. Bioact Mater.

[26] Seo Y, Bang S, Son J, Kim D, Jeong Y, Kim P, Kim HN (2021). Brain physiome: a concept bridging in vitro 3D brain models and in silico models for predicting drug toxicity in the brain. Bioact Mater.

[27] Xu G, Mahajan S, Roy I, Yong KT (2013). Theranostic quantum dots for crossing blood–brain barrier in vitro and providing therapy of HIV-associated encephalopathy. Front Pharmacol.

[28] Derk J, Jones HE, Como C, Pawlikowski B, Siegenthaler JA (2021). Living on the edge of the CNS: meninges cell diversity in health and disease. Front Cell Neurosci.

[29] Da Mesquita S, Fu Z, Kipnis J (2018). The meningeal lymphatic system: a new player in neurophysiology. Neuron.

[30] Zhao P, Le Z, Liu L, Chen Y (2020). Therapeutic delivery to the brain via the lymphatic vasculature. Nano Lett.

[31] Price RA, Johnson WW (1973). The central nervous system in childhood leukemia: I. Cancer.

[32] Ma Z, Zhao X, Huang J, Jia X, Deng M, Cui D, Xiao C (2017). A critical role of periostin in B-cell acute lymphoblastic leukemia. Leukemia.

[33] Erdener ŞE, Tang J, Kılıç K, Postnov D, Giblin JT, Kura S, Boas DA (2021). Dynamic capillary stalls in reperfused ischemic penumbra contribute to injury: a hyperacute role for neutrophils in persistent traffic jams. J Cereb Blood Flow Metab.

[34] Frishman-Levy L, Izraeli S (2017). Advances in understanding the pathogenesis of CNS acute lymphoblastic leukaemia and potential for therapy. Br J Haematol.

[35] Mullins N, Forstner AJ, O’Connell KS, Coombes B, Coleman JR, Qiao Z, Potash JB (2021). Genome-wide association study of more than 40,000 bipolar disorder cases provides new insights into the underlying biology. Nat Genet.

[36] Engelhardt B, Ransohoff RM (2012). Capture, crawl, cross: the T cell code to breach the blood–brain barriers. Trends Immunol.

[37] Yao Y, Chen ZL, Norris EH, Strickland S (2014). Astrocytic laminin regulates pericyte differentiation and maintains blood brain barrier integrity. Nat Commun.

[38] Gétaz EP, Miller GJ (1979). Spinal cord involvement in chronic lymphocytic leukemia. Cancer.

[39] Tavares GA, Louveau A (2021). Meningeal lymphatics: an immune gateway for the central nervous system. Cells.

[40] Yuan J, Li Y, Liu X, Nie M, Jiang W, Fan Y, Jiang R (2021). Atorvastatin plus low-dose dexamethasone may be effective for leukemia-related chronic subdural hematoma but not for leukemia encephalopathy: a report of three cases. Front Oncol.

[41] Olivier JC (2005). Drug transport to brain with targeted nanoparticles. NeuroRx.

[42] Parvez S, Kaushik M, Ali M, Alam MM, Ali J, Tabassum H, Kaushik P (2022). Dodging blood brain barrier with “nano” warriors: Novel strategy against ischemic stroke. Theranostics.

[43] Kreyling WG, Fertsch-Gapp S, Schäffler M, Johnston BD, Haberl N, Pfeiffer C, Parak WJ (2014). In vitro and in vivo interactions of selected nanoparticles with rodent serum proteins and their consequences in biokinetics. Beilstein J Nanotechnol.

[44] Zhou Q, Shao S, Wang J, Xu C, Xiang J, Piao Y, Shen Y (2019). Enzyme-activatable polymer–drug conjugate augments tumour penetration and treatment efficacy. Nat Nanotechnol.

[45] Mady OY, Donia AA, Al-Shoubki AA, Qasim W (2019). Paracellular pathway enhancement of metformin hydrochloride via molecular dispersion in span 60 microparticles. Front Pharmacol.

[46] Lochhead JJ, Yang J, Ronaldson PT, Davis TP (2020). Structure, function, and regulation of the blood-brain barrier tight junction in central nervous system disorders. Front Physiol.

[47] Sun Q, Leng J, Tang L, Wang L, Fu C (2021). A comprehensive review of the chemistry, pharmacokinetics, pharmacology, clinical applications, adverse events, and quality control of Indigo Naturalis. Front Pharmacol.

[48] Lin YC, Shih CP, Chen HC, Chou YL, Sytwu HK, Fang MC, Wang CH (2021). Ultrasound microbubble–facilitated inner ear delivery of gold nanoparticles involves transient disruption of the tight junction barrier in the round window membrane. Front Pharmacol.

[49] Subramanian, M. A. (2019). Toxicology: Principles and Methods. MJP Publisher.

[50] Kim GB, Aragon-Sanabria V, Randolph L, Jiang H, Reynolds JA, Webb BS, Dong C (2020). High-affinity mutant Interleukin-13 targeted CAR T cells enhance delivery of clickable biodegradable fluorescent nanoparticles to glioblastoma. Bioact Mater..

[51] Sanborn SL, Murugesan G, Marchant RE, Kottke-Marchant K (2002). Endothelial cell formation of focal adhesions on hydrophilic plasma polymers. Biomaterials.

[52] Banks WA (2012). Brain meets body: the blood-brain barrier as an endocrine interface. Endocrinology.

[53] Raj DS, Kesavan DK, Muthusamy N, Umamaheswari S (2021). Efflux pumps potential drug targets to circumvent drug Resistance-Multi drug efflux pumps of Helicobacter pylori. Materials Today: Proceedings.

[54] Huber JD, Egleton RD, Davis TP (2001). Molecular physiology and pathophysiology of tight junctions in the blood–brain barrier. Trends Neurosci.

[55] Soudmand P, Tofighi A, Azar JT, Razi M, Pakdel FG (2021). Different continuous exercise training intensities induced effect on sertoli-germ cells metabolic interaction; implication on GLUT-1, GLUT-3 and MCT-4 transporting proteins expression level. Gene.

[56] Hawkins RA, Viña JR, Mokashi A, Peterson DR, O’Kane R, Simpson IA, Rasgado-Flores H (2013). Synergism between the two membranes of the blood-brain barrier: Glucose and amino acid transport. Am J Neurosci Res..

[57] Correia AC, Monteiro AR, Silva R, Moreira JN, Lobo JS, Silva AC (2022). Lipid nanoparticles strategies to modify pharmacokinetics of central nervous system targeting drugs: crossing or circumventing the blood-brain barrier (BBB) to manage neurological disorders. Adv Drug Deliv Rev.

[58] Boveri M, Berezowski V, Price A, Slupek S, Lenfant AM, Benaud C, Dehouck MP (2005). Induction of blood-brain barrier properties in cultured brain capillary endothelial cells: comparison between primary glial cells and C6 cell line. Glia.

[59] Cox A, Andreozzi P, Dal Magro R, Fiordaliso F, Corbelli A, Talamini L, Chinello C, Raimondo F, Magni F, Tringali M, Krol S, Silva PJ, Stellacci F, Masserini M, Re F (2018). Evolution of nanoparticle protein corona across the blood–brain barrier. ACS nano..

[60] Piddock LJ (2006). Multidrug-resistance efflux pumps? not just for resistance. Nat Rev Microbiol.

[61] Lorke DE, Kalasz H, Petroianu GA, Tekes K (2008). Entry of oximes into the brain: a review. Curr Med Chem.

[62] Kuldo JM, Ogawara KI, Werner N, Ásgeirsdóttir SA, Kamps JA, Kok RJ, Molema G (2005). Molecular pathways of endothelial cell activation for (targeted) pharmacological intervention of chronic inflammatory diseases. Curr Vasc Pharmacol.

[63] Eilenberger C, Rothbauer M, Selinger F, Gerhartl A, Jordan C, Harasek M, Ertl P (2021). A microfluidic multisize spheroid array for multiparametric screening of anticancer drugs and blood–brain barrier transport properties. Adv Sci.

[64] Fernández L, Hancock RE (2012). Adaptive and mutational resistance: role of porins and efflux pumps in drug resistance. Clin Microbiol Rev.

[65] Kevadiya BD, Ottemann BM, Thomas MB, Mukadam I, Nigam S, McMillan J, Gendelman HE (2019). Neurotheranostics as personalized medicines. Adv Drug Deliv Rev.

[66] Liu L, Chen Q, Wen L, Li C, Qin H, Xing D (2019). Photoacoustic therapy for precise eradication of glioblastoma with a tumor site blood–brain barrier permeability upregulating nanoparticle. Adv Func Mater.

[67] Barbara R, Belletti D, Pederzoli F, Masoni M, Keller J, Ballestrazzi A, Grabrucker AM (2017). Novel Curcumin loaded nanoparticles engineered for Blood-Brain barrier crossing and able to disrupt Abeta aggregates. Int J Pharm.

[68] Hu Q, Gao X, Gu G, Kang T, Tu Y, Liu Z, Chen J (2013). Glioma therapy using tumor homing and penetrating peptide-functionalized PEG–PLA nanoparticles loaded with paclitaxel. Biomaterials.

[69] Zhao Y, Jiang Y, Lv W, Wang Z, Lv L, Wang B, Gu Z (2016). Dual targeted nanocarrier for brain ischemic stroke treatment. J Control Release.

[70] Thanh DTM, Trang PTT, Huong HT, Nam PT, Phuong NT, Trang NTT, Seo-Park J (2015). Fabrication of poly (lactic acid)/hydroxyapatite (PLA/HAp) porous nanocomposite for bone regeneration. Int J Nanotechnol.

[71] Anand P, O’Neil A, Lin E, Douglas T, Holford M (2015). Tailored delivery of analgesic ziconotide across a blood brain barrier model using viral nanocontainers. Sci Rep.

[72] Kumari S, Ahsan SM, Kumar JM, Kondapi AK, Rao NM (2017). Overcoming blood brain barrier with a dual purpose Temozolomide loaded Lactoferrin nanoparticles for combating glioma (SERP-17-12433). Sci Rep.

[73] Miao YB, Chen KH, Chen CT, Mi FL, Lin YJ, Chang Y, Sung HW (2021). A noninvasive gut-to-brain oral drug delivery system for treating brain tumors. Adv Mater.

[74] Elzoghby AO, Abd-Elwakil MM, Abd-Elsalam K, Elsayed TM, Hashem Y, Mohamed O (2016). Natural polymeric nanoparticles for brain-targeting: implications on drug and gene delivery. Current Pharm Des..

[75] Bala I, Hariharan S, Kumar MR (2004). PLGA nanoparticles in drug delivery: the state of the art. Crit Rev Ther Drug Carrier Syst.

[76] Bhowmik A, Chakravarti S, Ghosh A, Shaw R, Bhandary S, Bhattacharyya S, Ghosh MK (2017). Anti-SSTR2 peptide based targeted delivery of potent PLGA encapsulated 3, 3’-diindolylmethane nanoparticles through blood brain barrier prevents glioma progression. Oncotarget.

[77] Sun T, Zhang YS, Pang B, Hyun DC, Yang M, Xia Y, Voliani V (2021). Engineered nanoparticles for drug delivery in cancer therapy. Nanomaterials and Neoplasms.

[78] Thananukul K, Kaewsaneha C, Opaprakasit P, Lebaz N, Errachid A, Elaissari A (2021). Smart gating porous particles as new carriers for drug delivery. Adv Drug Deliv Rev.

[79] Muniswamy VJ, Raval N, Gondaliya P, Tambe V, Kalia K, Tekade RK (2019). ‘Dendrimer-Cationized-Albumin’encrusted polymeric nanoparticle improves BBB penetration and anticancer activity of doxorubicin. Int J Pharm.

[80] Florendo M, Figacz A, Srinageshwar B, Sharma A, Swanson D, Dunbar GL, Rossignol J (2018). Use of polyamidoamine dendrimers in brain diseases. Molecules.

[81] Liu Y, Alahiri M, Ulloa B, Xie B, Sadiq SA (2018). Adenosine A2A receptor agonist ameliorates EAE and correlates with Th1 cytokine-induced blood brain barrier dysfunction via suppression of MLCK signaling pathway. Immunity, Inflammation and Disease.

[82] Ding S, Khan AI, Cai X, Song Y, Lyu Z, Du D, Lin Y (2020). Overcoming blood–brain barrier transport: Advances in nanoparticle-based drug delivery strategies. Mater Today.

[83] Hong CS, Sharma P, Yerneni SS, Simms P, Jackson EK, Whiteside TL, Boyiadzis M (2017). Circulating exosomes carrying an immunosuppressive cargo interfere with cellular immunotherapy in acute myeloid leukemia. Sci Rep.

[84] Li H, Wang Y, Tang Q, Yin D, Tang C, He E, Peng Q (2021). The protein corona and its effects on nanoparticle-based drug delivery systems. Acta Biomater.

[85] Lee M, Li W, Siu RK, Whang J, Zhang X, Soo C, Wu BM (2009). Biomimetic apatite-coated alginate/chitosan microparticles as osteogenic protein carriers. Biomaterials.

[86] Caprifico AE, Foot PJ, Polycarpou E, Calabrese G (2020). Overcoming the blood-brain barrier: Functionalised chitosan nanocarriers. Pharmaceutics.

[87] Chipaux M, van der Laan KJ, Hemelaar SR, Hasani M, Zheng T, Schirhagl R (2018). Nanodiamonds and their applications in cells. Small.

[88] Bitounis D, Fanciullino R, Iliadis A, Ciccolini J (2012). Optimizing druggability through liposomal formulations: new approaches to an old concept. ISRN.

[89] Gaillard PJ, Appeldoorn CC, Dorland R, van Kregten J, Manca F, Vugts DJ, van Tellingen O (2014). Pharmacokinetics, brain delivery, and efficacy in brain tumor-bearing mice of glutathione pegylated liposomal doxorubicin (2B3–101). PLoS ONE.

[90] Wadajkar AS, Dancy JG, Hersh DS, Anastasiadis P, Tran NL, Woodworth GF, Kim AJ (2017). Tumor-targeted nanotherapeutics: overcoming treatment barriers for glioblastoma. Wiley Interdisciplinary Rev Nanomed Nanobiotechnol..

[91] Mulvihill JJ, Cunnane EM, Ross AM, Duskey JT, Tosi G, Grabrucker AM (2020). Drug delivery across the blood–brain barrier: recent advances in the use of nanocarriers. Nanomedicine.

[92] Gao H, Yang Z, Zhang S, Cao S, Shen S, Pang Z, Jiang X (2013). Ligand modified nanoparticles increases cell uptake, alters endocytosis and elevates glioma distribution and internalization. Sci Rep.

[93] Wu S, Fu J, Liu D, Chen D, Hu H (2020). The Blood-Brain Barrier Cell-Targeted Gene Delivery System to Enhance Nerve Growth Factor Protein Secretion in the Brain. ACS Biomater Sci Eng.

[94] Mitchell MJ, Billingsley MM, Haley RM, Wechsler ME, Peppas NA, Langer R (2021). Engineering precision nanoparticles for drug delivery. Nat Rev Drug Discovery.

[95] Wei J, Wang Y, Jiang J, Yan Y, Fan D, Yang X, Li J (2019). Development of an antibacterial bone graft by immobilization of levofloxacin hydrochloride-loaded mesoporous silica microspheres on a porous scaffold surface. J Biomed Nanotechnol.

[96] Kuang J, Song W, Yin J, Zeng X, Han S, Zhao YP, Zhang XZ (2018). iRGD modified chemo-immunotherapeutic nanoparticles for enhanced immunotherapy against glioblastoma. Adv Functional Mater..

[97] Yin T, Xie W, Sun J, Yang L, Liu J (2016). Penetratin peptide-functionalized gold nanostars: enhanced BBB permeability and NIR photothermal treatment of Alzheimer’s disease using ultralow irradiance. ACS Appl Mater Interfaces.

[98] Chen IC, Hsiao IL, Lin HC, Wu CH, Chuang CY, Huang YJ (2016). Influence of silver and titanium dioxide nanoparticles on in vitro blood-brain barrier permeability. Environ Toxicol Pharmacol.

[99] Rivera-Gil P, De Jimenez Aberasturi D, Wulf V, Pelaz B, Del Pino P, Zhao Y, Parak WJ (2013). The challenge to relate the physicochemical properties of colloidal nanoparticles to their cytotoxicity. Acc Chem Res.

[100] Norek M, Pereira GA, Geraldes CF, Denkova A, Zhou W, Peters JA (2007). NMR transversal relaxivity of suspensions of lanthanide oxide nanoparticles. The Journal of Physical Chemistry C.

[101] Cheng Y, Morshed RA, Auffinger B, Tobias AL, Lesniak MS (2014). Multifunctional nanoparticles for brain tumor imaging and therapy. Adv Drug Deliv Rev.

[102] Raman S, Mahmood S, Hilles AR, Javed MN, Azmana M, Al-Japairai KA (2020). Polymeric nanoparticles for brain drug delivery-a review. Curr Drug Metab.

[103] Moosavi MA, Sharifi M, Ghafary SM, Mohammadalipour Z, Khataee A, Rahmati M, Ghavami S (2016). Photodynamic N-TiO_2_ nanoparticle treatment induces controlled ROS-mediated autophagy and terminal differentiation of leukemia cells. Sci Rep.

[104] Choy JH, Choi SJ, Oh JM (2006). Cellular uptake mechanism of an inorganic nanovehicle and its drug conjugates: enhanced efficacy due to clathrin-mediated endocytosis. Bioconjugate Chem..

[105] Prokop A, Davidson JM (2008). Nanovehicular intracellular delivery systems. J Pharm Sci.

[106] Salatin S, Maleki Dizaj S, Yari Khosroushahi A (2015). Effect of the surface modification, size, and shape on cellular uptake of nanoparticles. Cell Biol Int.

[107] Khine YY, Stenzel MH (2020). Surface modified cellulose nanomaterials: a source of non-spherical nanoparticles for drug delivery. Mater Horiz.

[108] Pitirollo O, Micoli F, Necchi F, Mancini F, Carducci M, Adamo R, Lay L (2020). Gold nanoparticles morphology does not affect the multivalent presentation and antibody recognition of Group A Streptococcus synthetic oligorhamnans. Bioorg Chem.

[109] Gonzalez-Carter DA, Ong ZY, McGilvery CM, Dunlop IE, Dexter DT, Porter AE (2019). L-DOPA functionalized, multi-branched gold nanoparticles as brain-targeted nano-vehicles. Nanomedicine.

[110] Alexis F, Pridgen E, Molnar LK, Farokhzad OC (2008). Factors affecting the clearance and biodistribution of polymeric nanoparticles. Mol Pharm.

[111] Zhang B, Sun X, Mei H, Wang Y, Liao Z, Chen J, Jiang X (2013). LDLR-mediated peptide-22-conjugated nanoparticles for dual-targeting therapy of brain glioma. Biomaterials.

[112] Brunacci N, Neffe AT, Wischke C, Naolou T, Nöchel U, Lendlein A (2019). Oligodepsipeptide (nano) carriers: Computational design and analysis of enhanced drug loading. J Control Release.

[113] Jackson AW, Fulton DA (2013). Making polymeric nanoparticles stimuli-responsive with dynamic covalent bonds. Polym Chem.

[114] Tan C, Arshadi M, Lee MC, Godec M, Azizi M, Yan B, Abbaspourrad A (2019). A robust aqueous core–shell–shell coconut-like nanostructure for stimuli-responsive delivery of hydrophilic cargo. ACS Nano.

[115] Zhang W, Zhang Z, Zhang Y (2011). The application of carbon nanotubes in target drug delivery systems for cancer therapies. Nanoscale Res Lett.

[116] Tanner P, Baumann P, Enea R, Onaca O, Palivan C, Meier W (2011). Polymeric vesicles: from drug carriers to nanoreactors and artificial organelles. Acc Chem Res.

[117] Tang L, Zhao CY, Wang XH, Li RS, Yang JR, Huang YP, Liu ZS (2015). Macromolecular crowding of molecular imprinting: a facile pathway to produce drug delivery devices for zero-order sustained release. Int J Pharm.

[118] Chan JM, Valencia PM, Zhang L, Langer R, Farokhzad OC, Grobmyer SR, Moudgil BM (2010). Polymeric nanoparticles for drug delivery. Cancer Nanotechnology.

[119] Wheeler KE, Chetwynd AJ, Fahy KM, Hong BS, Tochihuitl JA, Foster LA, Lynch I (2021). Environmental dimensions of the protein corona. Nat Nanotechnol.

[120] Shubar HM, Dunay IR, Lachenmaier S, Dathe M, Bushrab FN, Mauludin R, Liesenfeld O (2009). The role of apolipoprotein E in uptake of atovaquone into the brain in murine acute and reactivated toxoplasmosis. J Drug Target.

[121] Cai R, Chen C (2019). The crown and the scepter: roles of the protein corona in nanomedicine. Adv Mater.

[122] Lipka J, Semmler-Behnke M, Sperling RA, Wenk A, Takenaka S, Schleh C, Kreyling WG (2010). Biodistribution of PEG-modified gold nanoparticles following intratracheal instillation and intravenous injection. Biomaterials.

[123] Šamec N, Zottel A, Videtič Paska A, Jovčevska I (2020). Nanomedicine and immunotherapy: a step further towards precision medicine for glioblastoma. Molecules.

[124] Liu L, Xu K, Wang H, Jeremy Tan PK, Fan W, Venkatraman SS, Yang YY (2009). Self-assembled cationic peptide nanoparticles as an efficient antimicrobial agent. Nature Nanotechnol.

[125] Choi CHJ, Alabi CA, Webster P, Davis ME (2010). Mechanism of active targeting in solid tumors with transferrin-containing gold nanoparticles. Proc Natl Acad Sci.

[126] Johnsen KB, Bak M, Melander F, Thomsen MS, Burkhart A, Kempen PJ, Moos T (2019). Modulating the antibody density changes the uptake and transport at the blood-brain barrier of both transferrin receptor-targeted gold nanoparticles and liposomal cargo. J Control Release.

[127] Zhang C, Wan X, Zheng X, Shao X, Liu Q, Zhang Q, Qian Y (2014). Dual-functional nanoparticles targeting amyloid plaques in the brains of Alzheimer’s disease mice. Biomaterials.

[128] Belhadj Z, Ying M, Cao X, Hu X, Zhan C, Wei X, Lu W (2017). Design of Y-shaped targeting material for liposome-based multifunctional glioblastoma-targeted drug delivery. J Control Release.

[129] Zhang J, Hu K, Di L, Wang P, Liu Z, Zhang J, Qiao H (2021). Traditional herbal medicine and nanomedicine: Converging disciplines to improve therapeutic efficacy and human health. Adv Drug Deliv Rev.

[130] Miao YB, Ren HX, Zhong Q, Song FX (2022). Tailoring a luminescent metal− organic framework precise inclusion of Pt-Aptamer nanoparticle for noninvasive monitoring Parkinson's disease. Chem Eng J.

[131] Kozlovskaya L, Abou-Kaoud M, Stepensky D (2014). Quantitative analysis of drug delivery to the brain via nasal route. J Control Release.

[132] Alvarez-Erviti L, Seow Y, Yin H, Betts C, Lakhal S, Wood MJ (2011). Delivery of siRNA to the mouse brain by systemic injection of targeted exosomes. Nat Biotechnol.

[133] Nagata T, Dwyer CA, Yoshida-Tanaka K, Ihara K, Ohyagi M, Kaburagi H, Yokota T (2021). Cholesterol-functionalized DNA/RNA heteroduplexes cross the blood–brain barrier and knock down genes in the rodent CNS. Nat Biotechnol.

[134] Rip J (2016). Liposome technologies and drug delivery to the CNS. Drug Discov Today Technol.

[135] Gernert M, Feja M (2020). Bypassing the Blood-Brain Barrier: Direct Intracranial Drug Delivery in Epilepsies. Pharmaceutics.

